# Supplementary material for: Influence of partial or total substitution of wheat flour and sunflower oil with Sacha inchi (Plukenetia volubilis L.) flour and oil on the quality and nutritional properties of cookies
Source: PLoS One. 2026 Jan 6;21(1):e0340190. doi: 10.1371/journal.pone.0340190 (PMC12774371; doi:10.1371/journal.pone.0340190)
Supplement: Attachment — Submitted filename: PONE-D-25-18191_reviewer (1).pdf [file pone.0340190.s001.pdf]

PLOS ONE

Influence of partial or total substitution of wheat flour and sunflower oil with Sacha inchi (Plukenetia volubilis L.) flour and oil on the quality and nutritional properties of cookies

--Manuscript Draft--

**Manuscript Number:**	PONE-D-25-18191
**Article Type:**	Research Article
**Full Title:**	Influence of partial or total substitution of wheat flour and sunflower oil with Sacha inchi (Plukenetia volubilis L.) flour and oil on the quality and nutritional properties of cookies
**Short Title:**	Sacha Inchi as a Wheat and Oil Substitute in Cookies: Quality and Nutrition Effects
**Corresponding Author:**	José M. Alvarez-Suarez<br>Universidad San Francisco de Quito - Campus Cumbaya: Universidad San Francisco de Quito<br>Quito , ECUADOR
**Keywords:**	Sacha inchi flour; by-product valorization; functional cookies; ingredient substitution; plant-based protein; omega-3 fatty acids source; wheat flour alternative; sustainable food innovation
**Abstract:**	The growing demand for healthier and more sustainable food products has driven the development of functional bakery items using alternative ingredients. This study evaluated the effects of partially or totally replacing wheat flour and sunflower oil with defatted Sacha inchi (Plukenetia volubilis L.) oil cake flour and Sacha inchi oil on the nutritional, physicochemical, and functional properties of cookies. Nine formulations were developed, including a control and eight experimental variants. Proximate analysis showed that Sacha inchi flour significantly increased protein (reaching 19.65%, more than double the control), fat, fiber (nearly six times higher than control), ash, and energy content, while reducing carbohydrate levels. Mineral analysis revealed substantially higher levels of calcium (10.8-fold increase), magnesium (10.2-fold increase), potassium (3-fold increase), phosphorus (5.4-fold increase), and zinc (4.6-fold increase) in cookies with Sacha inchi flour, although iron content decreased by 25%. A significant improvement in fatty acid composition was obtained according with the lipid profiling, with higher polyunsaturated fats—especially alpha-linolenic acid (omega-3)—and lower saturated fats in formulations containing Sacha inchi oil. Formulations with Sacha inchi ingredients also showed increased the polyphenol content (up to 1.46-fold increase) and antioxidant activity (up to 1.99-fold increase in FRAP assay), highlighting their functional potential. Texture analysis revealed that Sacha inchi flour reduced hardness, contributing to softer cookies (up to 5.8 times softer than control), while Sacha inchi oil increased hardness (up to 2.4 times firmer). In terms of geometry, full replacement of sunflower oil with Sacha inchi oil increased cookie diameter and spread ratio, whereas Sacha inchi flour reduced diameter and increased thickness, likely due to its fiber and protein content. Color parameters were also affected, suggesting interactions between lipid, protein, and fiber components during baking. Overall, the replacement of wheat flour and sunflower oil with Sacha inchi derivatives improved the nutritional value, antioxidant capacity, and textural and physical properties of cookies. These results support the potential of Sacha inchi oil and oilcake flour as valuable ingredients for developing sustainable and health-promoting baked goods, while contributing to the valorization of underutilized Amazonian crops and agro-industrial by-products.
**Order of Authors:**	Fernando E. Alejandro Ruiz
	Julio F. Ortega Jácome
	Margarita G. Chancay Pinargote
	José R. Mora
	Andrea C. Landázuri
	Paola Vásconez Duchicela
	Julio Vásconez Espinoza

Powered by Editorial Manager® and ProduXion Manager® from Aries Systems Corporation

	Pablo Beltrán-Ayala
	María J. Andrade-Cuvi
	José M. Alvarez-Suarez
**Additional Information:**	
**Question**	**Response**
**Financial Disclosure**<br><br>Enter a financial disclosure statement that describes the sources of funding for the work included in this submission. Review the submission guidelines for detailed requirements. View published research articles from *PLOS ONE* for specific examples.<br><br>This statement is required for submission and **will appear in the published article** if the submission is accepted. Please make sure it is accurate.<br><br>**Funded studies**<br>Enter a statement with the following details:<br>• Initials of the authors who received each award<br>• Grant numbers awarded to each author<br>• The full name of each funder<br>• URL of each funder website<br>• Did the sponsors or funders play any role in the study design, data collection and analysis, decision to publish, or preparation of the manuscript?<br><br>Did you receive funding for this work?	Yes
Please add funding details.<br>as follow-up to "**Financial Disclosure**<br><br>Enter a financial disclosure statement that describes the sources of funding for the work included in this submission. Review the submission guidelines for detailed requirements. View published research articles from *PLOS ONE* for specific examples.<br><br>This statement is required for submission and **will appear in the published article** if	This work was supported by Universidad San Francisco de Quito through an Interdisciplinary Grant (Project ID: 23218) awarded to José M. Alvarez-Suárez.

Powered by Editorial Manager® and ProduXion Manager® from Aries Systems Corporation

the submission is accepted. Please make sure it is accurate.<br><br>**Funded studies**<br>Enter a statement with the following details:<br>• Initials of the authors who received each award<br>• Grant numbers awarded to each author<br>• The full name of each funder<br>• URL of each funder website<br>• Did the sponsors or funders play any role in the study design, data collection and analysis, decision to publish, or preparation of the manuscript?<br><br>Did you receive funding for this work?"	
Please select the country of your main research funder (please select carefully as in some cases this is used in fee calculation).<br>as follow-up to "**Financial Disclosure**<br><br>Enter a financial disclosure statement that describes the sources of funding for the work included in this submission. Review the submission guidelines for detailed requirements. View published research articles from *PLOS ONE* for specific examples.<br><br>This statement is required for submission and **will appear in the published article** if the submission is accepted. Please make sure it is accurate.<br><br>**Funded studies**<br>Enter a statement with the following details:<br>• Initials of the authors who received each award<br>• Grant numbers awarded to each author<br>• The full name of each funder<br>• URL of each funder website<br>• Did the sponsors or funders play any role in the study design, data collection and analysis, decision to publish, or preparation of the manuscript?	ECUADOR - EC

Powered by Editorial Manager® and ProduXion Manager® from Aries Systems Corporation

Did you receive funding for this work?"	
**Competing Interests**<br><br>Use the instructions below to enter a competing interest statement for this submission. On behalf of all authors, disclose any competing interests that could be perceived to bias this work—acknowledging all financial support and any other relevant financial or non-financial competing interests.<br><br>This statement is required for submission and **will appear in the published article** if the submission is accepted. Please make sure it is accurate and that any funding sources listed in your Funding Information later in the submission form are also declared in your Financial Disclosure statement.<br><br>View published research articles from *PLOS ONE* for specific examples.<br><br>**NO authors have competing interests**<br><br>Enter: *The authors have declared that no competing interests exist.*<br><br>**Authors with competing interests**<br><br>Enter competing interest details beginning with this statement:<br><br>*I have read the journal's policy and the authors of this manuscript have the following competing interests: [insert competing interests here]*<br><br>* typeset	The authors have declared that no competing interests exist.
**Ethics Statement**<br><br>Enter an ethics statement for this submission. This statement is required if the study involved:<br><br>• Human participants<br>• Human specimens or tissue	N/A

Powered by Editorial Manager® and ProduXion Manager® from Aries Systems Corporation

• Vertebrate animals or cephalopods<br>• Vertebrate embryos or tissues<br>• Field research<br><br>Write "N/A" if the submission does not require an ethics statement.<br><br>General guidance is provided below. Consult the submission guidelines for detailed instructions. **Make sure that all information entered here is included in the Methods section of the manuscript.**	

Powered by Editorial Manager® and ProduXion Manager® from Aries Systems Corporation

**Format for specific study types**<br><br>**Human Subject Research (involving human participants and/or tissue)**<br>• Give the name of the institutional review board or ethics committee that approved the study<br>• Include the approval number and/or a statement indicating approval of this research<br>• Indicate the form of consent obtained (written/oral) or the reason that consent was not obtained (e.g. the data were analyzed anonymously)<br><br>**Animal Research (involving vertebrate animals, embryos or tissues)**<br>• Provide the name of the Institutional Animal Care and Use Committee (IACUC) or other relevant ethics board that reviewed the study protocol, and indicate whether they approved this research or granted a formal waiver of ethical approval<br>• Include an approval number if one was obtained<br>• If the study involved *non-human primates*, add *additional details* about animal welfare and steps taken to ameliorate suffering<br>• If anesthesia, euthanasia, or any kind of animal sacrifice is part of the study, include briefly which substances and/or methods were applied<br><br>**Field Research**<br><br>Include the following details if this study involves the collection of plant, animal, or other materials from a natural setting:<br>• Field permit number<br>• Name of the institution or relevant body that granted permission	
**Data Availability**<br><br>Authors are required to make all data underlying the findings described fully available, without restriction, and from the time of publication. PLOS allows rare exceptions to address legal and ethical concerns. See the PLOS Data Policy and FAQ for detailed information.	Yes - all data are fully available without restriction

Powered by Editorial Manager® and ProduXion Manager® from Aries Systems Corporation

A Data Availability Statement describing where the data can be found is required at submission. Your answers to this question constitute the Data Availability Statement and **will be published in the article**, if accepted.<br><br>**Important:** Stating 'data available on request from the author' is not sufficient. If your data are only available upon request, select 'No' for the first question and explain your exceptional situation in the text box.<br><br>Do the authors confirm that all data underlying the findings described in their manuscript are fully available without restriction?	
**Describe where the data may be found in full sentences. If you are copying our sample text, replace any instances of XXX with the appropriate details.**<br><br>• If the data are **held or will be held in a public repository**, include URLs, accession numbers or DOIs. If this information will only be available after acceptance, indicate this by ticking the box below. For example: *All XXX files are available from the XXX database (accession number(s) XXX, XXX.*).<br>• If the data are all contained **within the manuscript and/or Supporting Information files**, enter the following: *All relevant data are within the manuscript and its Supporting Information files.*<br>• If neither of these applies but you are able to provide **details of access elsewhere**, with or without limitations, please do so. For example:<br><br>*Data cannot be shared publicly because of [XXX]. Data are available from the XXX Institutional Data Access / Ethics Committee (contact via XXX) for researchers who meet the criteria for access to confidential data.*<br><br>*The data underlying the results presented in the study are available from (include the name of the third party*	All relevant data are within the manuscript and its Supporting Information files.

and contact information or URL).<br>• This text is appropriate if the data are owned by a third party and authors do not have permission to share the data.<br><br>* typeset	
Additional data availability information:	

Powered by Editorial Manager® and ProduXion Manager® from Aries Systems Corporation

Manuscript

Click here to access/download;Manuscript;Alvarez-Suarez_JM_et_al_MAIN TETX_rev.docx

Influence of partial or total substitution of wheat flour and sunflower oil with Sacha inchi (*Plukenetia volubilis* L.) flour and oil on the quality and nutritional properties of cookies

Fernando E. Alejandro Ruiz[1†], Julio F. Ortega Jácome[1†], Margarita G. Chancay Pinargote[1], José R. Mora[2], Andrea C. Landázuri[3,4,5,6], Paola Vásconez Duchicela[7], Julio Vásconez Espinoza[7], Pablo Beltrán-Ayala[8], María J. Andrade-Cuvi[1*], José M. Alvarez-Suarez[1,9*]

[1]Laboratorio de Investigación en Ingeniería en Alimentos (LabInAli), Departamento de Ingeniería en Alimentos, Colegio de Ciencias e Ingenierías, Universidad San Francisco de Quito USFQ, Quito 170901, Ecuador

[2]Departamento de Ingeniería Química, Colegio de Ciencias e Ingenierías, Universidad San Francisco de Quito USFQ, Quito 170901, Ecuador

[3]Chemical Engineering Department, Applied Circular Engineering & Simulation Group (GICAS), Universidad San Francisco de Quito USFQ, Quito 170901, Ecuador

[4]IBioMed, Universidad San Francisco de Quito USFQ, Quito 170901, Ecuador

[5]Institute for Energy and Materials, Universidad San Francisco de Quito USFQ, Quito 170901, Ecuador

[6]Instituto de Investigaciones Biológicas y Ambientales (Biósfera), Universidad San Francisco de Quito USFQ, Quito 170901, Ecuador

[7]Instituto Superior Universitario Oriente ITSO, Joya de los Sachas, Orellana 220101, Ecuador

[8]School of Economics, Universidad San Francisco de Quito USFQ, Quito 170901, Ecuador

[9]Laboratorio de Bioexploración, Colegio de Ciencias Biológicas y Ambientales, Universidad San Francisco de Quito USFQ, Quito 170901, Ecuador

*** Correspondence:**

María J. Andrade-Cuvi

mjandrade@usfq.edu.ec

José M. Alvarez-Suarez

jalvarez@usfq.edu.ec

† The authors contributed equally to the present research.

Abstract

The growing demand for healthier and more sustainable food products has driven the development of functional bakery items using alternative ingredients. This study evaluated the effects of partially or totally replacing wheat flour and sunflower oil with defatted Sacha inchi (*Plukenetia volubilis* L.) oil cake flour and Sacha inchi oil on the nutritional, physicochemical, and functional properties of cookies. Nine formulations were developed, including a control and eight experimental variants. Proximate analysis showed that Sacha inchi flour significantly increased protein (reaching 19.65%, more than double the control), fat, fiber (nearly six times higher than control), ash, and energy content, while reducing carbohydrate levels. Mineral analysis revealed substantially higher levels of calcium (10.8-fold increase), magnesium (10.2-fold increase), potassium (3-fold increase), phosphorus (5.4-fold increase), and zinc (4.6-fold increase) in cookies with Sacha inchi flour, although iron content decreased by 25%. A significant improvement in fatty acid composition was obtained according with the lipid profiling, with higher polyunsaturated fats—especially alpha-linolenic acid (omega-3)—and lower saturated fats in formulations containing Sacha inchi oil. Formulations with Sacha inchi ingredients also showed increased the polyphenol content (up to 1.46-fold increase) and antioxidant activity (up to 1.99-fold increase in FRAP assay), highlighting their functional potential. Texture analysis revealed that Sacha inchi flour reduced hardness, contributing to softer cookies (up to 5.8 times softer than control), while Sacha inchi oil increased hardness (up to 2.4 times firmer). In terms of geometry, full replacement of sunflower oil with Sacha inchi oil increased cookie diameter and spread ratio, whereas Sacha inchi flour reduced diameter and increased thickness, likely due to its fiber and protein content. Color parameters were also affected, suggesting interactions between lipid, protein, and fiber components during baking. Overall, the replacement of wheat flour and sunflower oil with Sacha inchi derivatives improved the nutritional value, antioxidant capacity, and textural and physical properties of cookies. These results support the potential of Sacha inchi oil and oilcake flour

as valuable ingredients for developing sustainable and health-promoting baked goods, while contributing to the valorization of underutilized Amazonian crops and agro-industrial by-products.

Keywords: Sacha inchi flour, by-product valorization, functional cookies, ingredient substitution, plant-based protein, omega-3 fatty acids source, wheat flour alternative, sustainable food innovation

Introduction

Commented [T1]: In view of this manuscript, the data given about Sacha inch is not sufficient. Give some detail about this composition, etc.

In recent years, the demand for nutritionally enhanced and functionally innovative food products has grown significantly, driven by increased consumer awareness of healthier diets, environmental sustainability, and the need for greater food system diversification [1]. This trend has motivated the food industry to explore the development of alternative formulations that not only address health-related concerns but also support sustainable production practices. Consumers now increasingly seek foods that offer functional benefits, such as improved digestive health, cardiovascular protection, or enhanced protein content, while also contributing to ethical and environmentally responsible consumption patterns. The baking industry, which has traditionally relied on refined wheat flour and conventional vegetable oils, now faces multiple challenges, including the prevalence of gluten intolerance, rising cases of metabolic disorders, and the environmental impact of monoculture-based grain production [2]. In response, researchers and manufacturers are investigating the substitution of traditional ingredients with alternative raw materials that meet modern nutritional expectations and align with broader goals of environmental sustainability and circular economy principles [3]. In this context, there is growing interest in the use of non-conventional flours and oils, derived from plant-based sources, agro-industrial by-products, or underutilized crops, as promising candidates for innovation in baked goods.

One promising approach involves the recovery and revalorization of by-products from the food industry to develop new formulations with higher nutritional value and reduced environmental impact. This aligns with the principles of the circular economy, which seeks to minimize waste and promote

the efficient use of resources throughout the production chain [4]. By turning food waste and processing residues into valuable functional ingredients, the food industry can improve product sustainability and contribute to closing resource loops. In particular, exotic plant-based ingredients, often rich in bioactive compounds, have gained attention due to their unique nutritional, functional, and sensory properties [5]. Many of these novel ingredients originate from biodiverse ecosystems, such as the Amazon rainforest, and have been traditionally consumed by indigenous communities for their health benefits and nutrient density. Their incorporation into modern food systems not only offers sustainable alternatives to conventional ingredients, but also supports the economic valorization of native crops, encourages agro-biodiversity, and helps reduce dependence on resource-intensive monocultures [6]. These strategies contribute to multiple Sustainable Development Goals (SDGs), particularly those related to responsible consumption and production, good health and well-being, and climate action. Among these emerging ingredients, Sacha inchi (*Plukenetia volubilis* L.), a plant native to the Amazon basin and traditionally cultivated by indigenous communities, has attracted significant scientific and commercial interest. It is particularly recognized for its remarkable protein and lipid profile, including high levels of omega-3 fatty acids, essential amino acids, and micronutrients [7]. Among the various products derived from this plant, one of the most prominent is Sacha inchi oil, which has been widely commercialized for its exceptional fatty acid composition and associated health benefits. However, the defatted seed residue, commonly known as Sacha inchi oil cake, remains an underutilized by-product, despite being rich in protein, dietary fiber, and essential minerals. This by-product offers significant potential for use in food formulations as a partial or total substitute for wheat flour, thus improving the overall nutritional value of baked products [8–10]. It is important to highlight that some reports in the literature support the idea that both the oil and the flour obtained from sacha inchi are non-toxic, suggesting that their consumption is safe and potentially beneficial for food formulation, provided that the seed flour undergoes appropriate thermal treatment [10,11].

Despite the promising nutritional profile of Sacha inchi, several practical challenges limit the commercial utilization of its by-products, including inconsistent extraction efficiency, underdeveloped supply chains and potential cost disadvantages compared to conventional ingredients. Nevertheless, cookies represent an ideal vehicle for ingredient substitution due to their widespread acceptance across age groups. While interest in Sacha inchi as a functional ingredient is growing, research examining the simultaneous application of both its flour and oil as direct replacements for traditional baking ingredients remains limited, creating a significant gap in understanding their combined effects in eveyday food products.

Against this background, our study evaluated how replacing wheat flour and sunflower oil with Sacha inchi derivatives affects cookies' nutritional profile, functional properties, and physical characteristics. We conducted comprehensive bromatological analyses to determine changes in key nutrients (protein, fat, fiber, carbohydrates, and energy content) and examined macro- and micronutrient composition to assess the nutritional impact of these substitutions. Aditionally, we analyzed fatty acid profiles to evaluate potential cardioprotective benefits from Sacha inchi oil incorporation, and measured polyphenol content and antioxidant capacity to quantify the functional properties these Amazonian ingredients contribute to the final products.

Materials and methods

Materials

The raw materials for cookie production were sourced from local supermarkets in Quito, Ecuador. The Sacha inchi (*Plukenetia volubilis* L.) defatted cake and oil were donated by members of the Shushufindi community (0.1883° S, 76.6422° W), located in the northern Amazon region of Ecuador, in the province of Sucumbíos. To extract the oil and obtain the defatted oil cake, the peeled Sacha inchi seeds were subjected to oil extraction by cold extrusion at room temperature, which allowed the crude oil to be separated from the defatted solid residue (oil cake). The resulting oil was left to stand for 24

hours in an airtight container, protected from light, at room temperature to promote the settling of suspended particles. After decantation, the clarified upper layer of oil was filtered through a linen cloth and stored in airtight green glass bottles at 4°C. The chemical and bromatological composition of the raw materials (defatted sacha inchi cake flour and Sacha inchi oil) is available in the supplementary materials for reference. Both the Sacha inchi cake flour and the cookies were ground into a fine powder using a RIRIHONG 2000A multifunction knife mill (Zhejiang, China) and stored at 4 °C in vacuum-sealed plastic bags until further use. The flour, obtained from the milling of the oil cake, was used in the cookie formulations, while the ground cookies were used for bromatological and chemical analyses.

Formulation of cookies

The cookies were made based on the method previously described by Auquiñivin & Castro [12]. In this study, nine sweet cookie formulations were developed: one control sample (Ctl), prepared using a traditional recipe with wheat flour and sunflower oil, and eight experimental formulations (F1–F8), in which wheat flour and/or sunflower oil were partially or completely replaced with sacha inchi oilcake flour and Sacha inchi oil. The level of substitution varied as follows: formulations F1–F2 included increasing levels of Sacha inchi oil (50% and 100%, respectively), while keeping the wheat flour unchanged; formulations F3–F5 involved a 50% replacement of wheat flour with oilcake flour combined with 0%, 50%, and 100% sacha inchi oil, respectively; and formulations F6–F8 consisted of a full (100%) replacement of wheat flour with oilcake flour, combined with increasing levels of Sacha inchi oil (0%, 50%, and 100%, respectively). The exact percentages of each ingredient are detailed in **Table 1.**

To make the cookies, the dry ingredients were mixed: wheat flour or/and Sacha inchi cake, sodium bicarbonate and salt, and kept at room temperature. Brown sugar, white sugar, and shortening and/or sacha inchi oil were mixed for 2 minutes using a Classic™ Max Watts 275 mixer (KitchenAid®, Michigan, USA). Then, the vanilla essence and honey were added and mixed for 2 more minutes. To this mixture the previously mixed dry ingredients were added, and everything was homogenized until

a homogeneous dough was obtained in a mixer bowl. Then, the dough was refrigerated at 4 ºC for 10 minutes. The prepared dough was divided into 5 g portions, which were placed on a previously greased oblong-shaped tray. The dough was rolled out by moving it back and forth once, to a thickness of 1 cm and shaped using moulds with a diameter of 4 cm. The cookies were then baked at 180 °C for 12 min using a commercial convection oven (Prática Technipan, Brazil). The baked cookies were allowed to cool for 30 min at room temperature and then wrapped in aluminium foil and kept at room temperature for 24 h until analysis.

Proximate analysis

Proximate composition analyses of pre-ground cookies were conducted following the standardized protocols outlined in the AOAC Official Methods of Analysis. Color was evaluated using a Konica Minolta CR-400 colorimeter (Konica Minolta Sensing Americas, Inc., NY, EEUU) in the CIELAB color space. Moisture content (%) was determined through gravimetric analysis [13], whereas water activity (a_w) was measured using a HygroLab C1 water activity meter (Rotronic, Grindelstrasse, CH-Switzerland). Ash content (%) was quantified by incinerating 3 g of samples in a muffle furnace at 550 ± 1 °C overnight [14]. Additionally, total crude fiber content (%) was assessed through sequential extraction with acidic and alkaline solutions [15] whyle the fraction corresponding to soluble (%) and insoluble fiber (%) using methods proposed by the Cereals & Grains Association [16] and AOAC [17]. Meanwhile, lipid content (%) was determined via Soxhlet extraction using petroleum ether as the solvent [18], whereas protein content (%) was analyzed using the Kjeldahl method, applying a nitrogen-to-protein conversion factor of 6.25 [19]. Finally, carbohydrate content (g/100 g) and energy value (kcal/100 g) were calculated using Equations (1) and (2), as described by Plustea et al. [20], respectively, as shown below:

Carbohydrate (g/100 g) = 100 – (fat + protein + water + ash) *(1)*

Energy value (kcal/100 g) = (fat *x* 9) + (carbohydrate *x* 4) + (protein *x* 4) *(2)*

Commented [T2]: Some common mistakes like incorrect spellings, grammatical mistakes are also present in this manuscript. Kindly correct all these.

Gas chromatography-mass spectrometry (GC-MS) analysis of fatty acid composition

Fatty acid content analysis was performed following the internal standard method described by Ponce et al. and based on AC Analytical Controls Application Note 1410 [21,22]. For this procedure, a transesterification reaction was first carried out on 0.5 ± 0.01 g of the oil extracted from the cookies, obtained by Soxhlet extraction with petroleum ether, using methanol (methanol: fat molar ratio: 14:1) and 1% m/m potassium hydroxide (KOH) as catalyst. To facilitate quantification, methyl dodecanoate (2.0 mg/mL) was used as an internal standard. The resulting fatty acid methyl esters (FAMEs) were dissolved in hexane before analysis.

For GC-MS analysis, a Shimadzu GCMS-QP-2010 Ultra Plus equipped with an AOC-20i/s autosampler and an autoinjector was used. The separation was performed on a DB-WAX gas chromatography (GC) capillary column (column thickness: 0.25 µm, length: 30.0 m, internal diameter: 0.25 µm). The oven configuration was set as follows: an initial temperature of 120 °C with a hold time of 2 min, followed by a ramp to 190 °C at 15 °C/min and a hold time of 4 min, then a ramp to 240 °C at 5 °C/min and a hold time of 10 min, and finally a ramp to 250 °C at 5 °C/min and a hold time of 3 min. The injector temperature was set at 250 °C, and 1 µL of the sample was injected with a 1:4 split mode. Helium was used as the carrier gas, considering the linear velocity for a column flow of 0.98. Mass detection was configured to start at an *m/z* of 35.00 and end at an *m/z* of 700.00. To establish the calibration curves, individual fatty acid standards (concentration range: 1.45–2.55 mg/L) were used for palmitic, oleic, and linoleic acids. Fatty acid quantification was based on a 1:1 response factor, meaning that the mass ratio (mi/mt) was equivalent to the area ratio (Ai/At), where the mass fraction of each fatty acid was determined using the equation:

$$\% \text{ Fai} = (\text{Ai}/\text{At})\ x\ 100 \qquad (3)$$

where % Fai is the percentage of the corresponding fatty acid "i", Ai is the area of the given fatty acid, and At is the total area (At = ΣAi). In the calibration curve equations, the mass ratio is "x", and

Commented [T3]: In GC MS Analysis, there is no citation reference is given in overall paragraph.

the area ratio is “y”. The results were expressed in grams of fatty acids per 100 g of oil (g/100 g for the oil).

Determination of macro- and microelements using inductively coupled plasma optical emission spectrometry (ICP-OES)

Prior to analysis, ground cookies samples were digested according to the procedures outlined in AOAC Official Method 2015.1, "Heavy Metals in Foods" [23]. In brief, 0.5 ± 0.1 g of each sample was placed in a digestion vessel, followed by the addition of 9 mL of nitric acid (TraceMetal Grade™, Fisher Chemical™) and 0.4 mL of hydrogen peroxide (30%, Fisher Chemical™). The digested solution was then filtered through a 0.45 µm PTFE membrane and diluted to a final volume of 50 mL with deionized water. For quality control purposes, a spiked sample was prepared using the same procedure, yielding recoveries between 84.70% and 99.96%. The determination of macro- and microelements was carried out using an ICP-OES system (iCAP™ 7400 Duo ICP-OES spectrometer, Thermo Scientific™, Germany), operated with Thermo Scientific™ Qtegra™ Intelligent Scientific Data Solution™ (ISDS) software. Quality assurance and control measures included the use of an ICP VIII multi-element standard solution for calibration curve generation, with results reported in mg/100 g dry weight (DW).

Determination of polyphenols and total antioxidant activity

To evaluate polyphenol content and total antioxidant activity, a hydroalcoholic extraction was conducted based on the methodology previously reported by Sokmen et al.[24]. In summary, 0.5 ± 0.01 g of ground cookies, previously defatted, was extracted with 5 mL of an HClcon/methanol/water mixture (1/80:19, *v/v*). The solution was stirred in the dark at room temperature for 2 hours using a magnetic stirrer. Afterward, the sample was centrifuged (HERMLE Z 206 A, Wehingen, DE) at 20°C for 10 minutes at 10,000 rpm, filtered through a Whatman® Grade 1 qualitative filter paper (Sigma Aldrich, St. Louis, MO, USA), and stored at –20 °C until further analysis.

The determination of total polyphenol content (TPC) was carried out spectrophotometrically using an i3 UV-VIS Spectrophotometer (Hanon Instruments, Shandong, China) following the Folin-

Ciocalteu method [25]. A standard curve was generated using gallic acid, and results were expressed as mg of gallic acid equivalents per 100 g of fresh weight (mg GAE/100 g FW) of cookies. On the other hand, total antioxidant capacity was simultaneously evaluated using two spectrophotometric methods: the Ferric Reducing Antioxidant Power (FRAP) assay [26] and the 2,2-Diphenyl-1-picrylhydrazyl (DPPH) assay [27]. Trolox was used as standard to establish the calibration curve for both assays, and the results were expressed as millimoles of Trolox equivalents (mM TEq) per gram of fresh weight (mM TEq/g FW) of cookie.

Physical analysis of cookies

Diameter (D), Thickness (T) and Spread ratio

Six cookies from each treatment group were measured using a vernier caliper with a precision of ± 0.02 mm, following the standard procedures outlined by the American Association of Cereal Chemists (AACC) [28]. The diameter (D) and thickness (T) of each cookie were recorded, and the mean values were calculated and expressed in centimeters (cm). To determine the spreading ratio, the average diameter was divided by the average thickness (D/T), as previously described by Korese et al. [29].

Color determination

Commented [T4]: Again, there is no citation is given in color determination method, hardness determination and statistical analysis. Give proper citation in over all manuscript.

The surface color of the cookies was measured using a colorimeter (CR-400, Konica Minolta Inc., Japan), which was previously calibrated with a standard white calibration plate under D65 illuminant conditions (Y = 80.1, x = 0.3219, y = 0.3394). Color measurements were recorded using the CIE L*a*b* color space, where L^* represents lightness, a^* indicates the red-green axis, and b^* represents the yellow-blue axis. For each treatment, a minimum of six cookie samples were analyzed to ensure measurement accuracy and reproducibility. The total color difference (ΔE) compared to the control formulation was calculated using the following equation:

$$\Delta L = \sqrt{(L^*_0 - L^*)^2 + (a^*_0 - a^*)^2 + (b^*_0 - b^*)^2} \quad (4)$$

where L^*_0, a^*_0 and b^*_0 are the color values of the control sample, and L^*, a^*, b^* correspond to the color values of the treated cookies. This parameter provides an objective quantification of perceptible color differences resulting from ingredient or processing variations.

Hardness

The texture analysis of cookies was determined by using texture analyzer (TX-700-UK02/2023, LAMY Rheology Instruments, France). Hardness of the cookies was evaluated by using a cylindrical probe (TX-CY2H355) at a speed of 2.5 mm/s and compression distance was set at 4 mm. The triggering force was 50 g using a load cell of 250 N**.** Data were recorded in a RheoTex Software and the results were expressed in Newtons (N).

Design of Experiment and Statistical Analysis

A completely randomized 3^2 factorial design was used to evaluate the effect of two factors: flour and sunflower oil, which were replaced with Sacha inchi oil cake and Sacha inchi oil, respectively. Each factor was tested at three levels (0%, 50%, and 100%). The design included two factors, three levels, and three replicates, resulting in 27 randomized experimental units. The experimental design and randomization were conducted using Minitab 19. The samples were analyzed in triplicate, and results were reported as mean ± standard deviation (SD). Data were analyzed by analysis of variance (ANOVA), and means were compared using Tukey's test at a significance level of 0.05, using INFOSTAT statistical software (Facultad de Ciencias Agropecuarias, Universidad Nacional de Córdoba, Argentina). The samples were analyzed in triplicate, and results were reported as mean ± standard deviation (SD).

Results and discussion

Bromatological analysis of cookies formulated with Sacha inchi flour and oil

The analysis of the proximal and chemical composition of cookies formulated by replacing wheat flour and sunflower oil with defatted Sacha inchi oil cake flour and Sacha inchi oil showed significant

changes in various compositional and functional parameters (**Table 2**). According to the results of the bromatological analysis, the moisture content increased significantly ($p \leq 0.05$) in formulations with higher percentages of Sacha inchi oil cake flour and oil, reaching an increase of up to 1.16 times in the formulation with 100% flour and oil replacement compared to the control cookie. However, no significant differences were observed among formulations with the same level of flour substitution but different levels of oil substitution. Given that Sacha inchi oil cake contains a residual oil content of up to 25%, the results suggest that an increase in the replacement percentage of wheat flour with Sacha inchi oil cake flour effectively influences the fat content of the cookies and, consequently, their ability to retain moisture. Regarding the water activity values, the results revealed a significant decrease ($p \leq 0.05$) as the replacement percentage of wheat flour and sunflower oil increased, compared to formulations with a lower degree of substitution. Similarly to moisture content, no significant differences were observed among formulations with the same level of flour substitution but different levels of oil substitution. These results suggest that a higher substitution of wheat flour and sunflower oil with Sacha inchi oilcake flour and Sacha inchi oil is suitable for preventing microbial growth and extending product stability. The behavior observed in both moisture content and water activity can be attributed to several physicochemical factors. A formulation with a higher fat content can retain more water due to physicochemical and structural mechanisms. Fat acts as a structuring agent, modifying water distribution within the food matrix and reducing its loss during baking and storage [30]. Furthermore, its hydrophobic nature allows the formation of a barrier that limits water evaporation, effectively retaining moisture within the product's structure[31]. Additionally, fat can interfere with protein network formation and coat starch granules, affecting water absorption and retention. Its presence lowers water activity by reducing free water mobility, thereby contributing to decreased evaporation. Finally, in high-fat products, the texture is generally softer and less porous, which makes water retention more effective compared to low-fat cookies, which tend to be drier and more porous, thus promoting moisture loss [30]. In line with the results described above, the ash content increased

significantly with flour substitution, reaching a maximum of 2.38% in the formulation with 100% substitution of wheat flour by Sacha inchi cake flour, with an increase of 2.27-fold with respect to the control sample (1.05%). These results are consistent with previous findings, where the incorporation of sacha inchi cake flour in the preparation of Peruvian traditional flatbread resulted in a significant increase in ash content in the sacha inchi flour-based formulations [9]. Similarly, the protein content showed a significant increase ($p \leq 0.05$) when wheat flour was substituted by Sacha inchi oil cake flour. The formulations with 100% flour substitution presented values of 19.34–19.65%, more than 2.2 times compared to 8.83% in the control sample. This increase is attributable to the high protein content of the defatted sacha inchi cake flour, in line with the results of the previous bromatological analysis of this flour (**SM_Table 1 in Supplementary Materials**), as well as with findings reported in the literatura [32,33]. This makes these cookies a potentially relevant source of plant-based protein. These findings are also consistent with previous reports on the effects of sacha inchi flour in bread substitutes [9]. Similar to the case of previous parameters within the groups with the same flour substitution, no statistically significant differences were observed between the different levels of oil substitution. This suggests that the Sacha inchi defatted cake flour is the main determining factor in the protein content. On the other hand, the fat content increased significantly with the substitution of wheat flour by Sacha inchi cake flour, representing a 1.81-fold increase in the formulation with 100% substitution, consistent with previous reports where the incorporation of sacha inchi flour similarly increased the fat content in the formulations [9]. In the treatments with the same percentage of flour substitution, an increase proportional to the level of oil substitution was observed, suggesting that the balance of Sacha inchi oil and defatted cake flour influences fat retention in the formulation. Another compositional result to highlight is the carbohydrate content in each of the formulations. In this case, carbohydrate content decreased with flour substitution, showing a 1.67-fold reduction, while the energy value increased in all formulations with flour and oil substitution, reaching a 1.15-fold increase in the 100% wheat flour substitution. These results are in line with previous findings on the use of sacha inchi flour in bakery

formulations, where similar trends in carbohydrate reduction and energy increase were observed [9]. This result, in turn, can be explained by the higher fat content in the formulations with sacha inchi oil cake flour substitution, as previously observed in the bromatological analysis of this flour (**SM_Table 1 in Supplementary Materials**), which contains a residual fat content of up to 25%. From the point of view of fiber contribution by the different formulations, an improvement in the content of this component was observed. Specifically, the total crude fiber content increased by up to 5.98 times compared to the control when 100% defatted sacha inchi oilcake flour was incorporated, consistent with previous reports on the use of defatted sacha inchi cake flour in baked products [9]. Insoluble fiber showed a significant increase, up to 70.2 times, which highlights the high fiber content in the defatted Sacha inchi oilcake flour. Significant differences were observed between the treatments with different flour substitution. Soluble fiber showed a more variable behavior, reaching up to 1.88 times more in formulations with the highest level of flour substitution, compared to the control. From a functional and nutritional perspective, the content and contribution of dietary fiber in foods is of great importance due to its beneficial effect on digestive and metabolic health. Insoluble fiber, which showed the greatest increase in these formulations, favors intestinal transit, prevents constipation and contributes to the health of the intestinal microbiome [34,35]. On the other hand, soluble fiber has the ability to retain water and form gels, which can positively influence the feeling of satiety and the regulation of glucose and cholesterol in the blood [36]. In addition, adequate consumption of dietary fiber has been associated with a reduced risk of chronic diseases such as type 2 diabetes, cardiovascular diseases and some types of cancer [37]. Therefore, the increase in fiber content in these formulations not only improves their nutritional value, but also enhances their functionality as a food with potential health benefits.

Fatty acid profile and content of cookies formulated with Sacha inchi flour and oil

The fatty acid composition of the formulated cookies varied significantly ($p \leq 0.05$) depending on the level of substitution with sacha inchi oil and oil cake flour (**Table 2**), consistent with previous

findings [9]. The results indicate that the incorporation of Sacha inchi oil and oil cake flour influenced the profiles of saturated fatty acids (SFA), monounsaturated fatty acids (MUFA), and polyunsaturated fatty acids (PUFA) in the final formulation.

The content of saturated fatty acids, mainly palmitic acid (C16:0) and stearic acid (C18:0), showed variations among formulations. Formulations with a higher proportion of Sacha inchi oil generally had lower levels of palmitic acid than the control formulation (without replacement, F1) ($p \leq 0.05$). This is consistent with the lower SFA content of Sacha inchi oil (**SM_Table 2 in Supplementary Materials**) compared to sunflower oil, which typically contains higher amounts of these fatty acids [38] The inclusion of oil cake flour did not significantly increase SFA content, suggesting that it retains only minimal amounts of these fatty acids. The stearic acid content also showed significant ($p \leq 0.05$) variations. Formulations with higher levels of Sacha inchi oil cake flour exhibited a significant reduction ($p \leq 0.05$) in this fatty acid compared to the control, except for formulation with 50 % substitution of flour and 100 % sunflower oil substitution, which showed the highest stearic acid content, possibly due to specific interactions during formulation. The oleic acid (C18:1) content varied significantly among formulations ($p \leq 0.05$) and was primarily influenced by the level of substitution with Sacha inchi oil. The control formulation, 0% substitution, exhibited the highest oleic acid content, which aligns with the natural composition of sunflower oil, known for its high oleic acid content [38]. In contrast, increased Sacha inchi oil substitution led to a significant ($p \leq 0.05$) reduction in oleic acid levels, with the lowest content observed in formulations with the highest sunflower oil substitution. This is expected since Sacha inchi oil contains lower proportions of this monounsaturated fatty acid (**SM_Table 2 in Supplementary Materials**) compared to sunflower oil. On the other hand, the incorporation of Sacha inchi cake flour alone did not significantly alter the oleic acid content, reinforcing the idea that the cake fraction retains only limited amounts of this fatty acid. Regarding polyunsaturated fatty acids (PUFAs), substitution with Sacha inchi oil significantly increased ($p \leq 0.05$) the linolenic acid (C18:3) content. The highest levels were observed in formulations with 100% Sacha

inchi oil replacement. This increase is consistent with the composition of Sacha inchi oil (46.92% linolenic acid) (**Table 2**), highlighting its potential as an excellent source of omega-3 fatty acids for functional food applicationsIn contrast, formulations with no sunflower oil substitution had elevated levels of linoleic acid (C18:2), which is abundant in sunflower oil [38]. Finally, formulations with higher levels of Sacha inchi oil showed a slight but significant ($p \leq 0.05$) reduction in linoleic acid. The inclusion of oilcake did not significantly contribute to the increase in linoleic acid levels, confirming that sunflower oil is the main source of this PUFA in the formulations.

The changes in fatty acid composition due to the inclusion of Sacha inchi oil and oilcake have significant nutritional implications. The increased omega-3 (linolenic acid) content in formulations with Sacha inchi oil is beneficial for cardiovascular health, as omega-3 fatty acids are known for their anti-inflammatory properties. They can help lower triglycerides, reduce the risk of arrhythmias, and slow plaque buildup. Additionally, omega-3 fatty acids have been shown to reduce cardiovascular mortality and improve cardiovascular outcomes[39] . Thus, the reduction in SFA and the increase in PUFA, particularly in formulations with Sacha inchi oil substitution, suggest that these formulations may provide a healthier lipid profile compared to traditional formulations based solely on sunflower oil and wheat flour.

Macro- and microelement content of cookies formulated with Sacha inchi flour and oil

The analysis of macro- and microelement contents in cookie formulations with different levels of wheat flour replacement by Sacha inchi flour and sunflower oil replacement by Sacha inchi oil reveals significant patterns in the mineral composition of the final product (**Table 2)**. Within the macroelements, the levels of calcium (Ca), magnesium (Mg), potassium (K), and phosphorus (P) increased significantly ($p \leq 0.05$) with the incorporation of Sacha inchi flour in the formulation. These results are consistent with the high content reported of theses macroelements in the Sacha inchi cake flour (**SM_Table 1 in Supplementary Materials**) and previous finding [32,33], which explains its increase as the proportion of Sacha inchi cake flour in the mix increases (**Table 2**). In fact, in

formulations without substitution, the Ca and Mg contents remained low (~15 mg/100 g), whereas in formulations with 50% Sacha inchi flour substitution, these values quintupled, reaching approximately 76–80 mg/100 g. This corresponds to an increase of 5.3-fold for calcium and 5-fold for magnesium compared to the control formulations (without substitution). In formulations with 100% substitution, the values exceeded 150 mg/100 g, representing an increase of 10.8-fold for calcium and 10.2-fold for magnesium relative to the non-substituted formulations. Potassium (K) followed a similar trend, with a progressive increase from approximately 80 mg/100 g in the formulations without substitution to values exceeding 180 mg/100 g in those with 50% substitution, representing an increase of 2.25-fold. In formulations with 100% substitution, potassium reached up to 240 mg/100 g, representing a 3-fold increase compared to formulations without Sacha inchi flour. Phosphorus (P) levels also exhibited a substantial increase with the replacement of wheat flour by Sacha inchi flour. In the formulations with the highest Sacha inchi flour content, phosphorus reached up to 404 mg/100 g (F9), representing a 5.4-fold increase compared to the non-substituted formulations. These results are consistent with the high content reported in the sacha inchi cake flour (**SM_Table 1 in Supplementary Materials**) and previous finding [32,33], suggesting that sacha inchi flour is a rich source of this essential mineral. In contrast, sodium (Na) levels remained stable across all formulations, with values close to 310 mg/100 g, indicating that the substitution of both flour and oil did not significantly affect the sodium content in the final product.

Regarding microelements, iron (Fe), unlike macrominerals, exhibited a significant decreasing ($p \leq 0.05$) trend with the progressive replacement of wheat flour by Sacha inchi oil cake flour. In formulations without substitution, iron content exceeded 2 mg/100 g, whereas in formulations with 50% and 100% substitution, the values declined to approximately 1.5 mg/100 g. This corresponds to a 25% reduction compared to the non-substituted formulation, suggesting that wheat flour was a richer source of iron than Sacha inchi flour. Zinc (Zn), on the other hand, exhibited an inverse trend to iron, showing a significant ($p \leq 0.05$) increase with the incorporation of Sacha inchi flour. In formulations

without substitution, zinc levels were around 0.5 mg/100 g, whereas in those with complete substitution, zinc levels reached up to 2.6 mg/100 g, representing a 4.6-fold increase compared to formulations without Sacha inchi flour. This finding suggests that Sacha inchi flour is a substantial source of zinc, an essential mineral involved in metabolic functions and immune system regulation.

The results indicate that the replacement of wheat flour with Sacha inchi cake flour and sunflower oil with Sacha inchi oil has a significant impact on the mineral profile of the cookies. The incorporation of Sacha inchi flour enhances the content of essential macrominerals such as calcium, magnesium, potassium, and phosphorus, which may offer nutritional benefits, particularly for populations at risk of deficiencies in these nutrients. However, the observed reduction in iron content suggests that additional sources of this mineral may be required to prevent potential deficiencies in the final product. From a technological perspective, the stability of sodium content and the increase in zinc levels may be advantageous, as zinc plays a critical role in enzymatic functions and protein structural stability. Moreover, the high phosphorus content could improve certain functional properties of the dough, such as water retention capacity and structural integrity of the final product.

Total polyphenol content and antioxidant capacity of cookies formulated with Sacha inchi flour and oil

The influence of different substitutions on the contribution of compounds with functional properties was analyzed. **Table 1** presents the results of the analysis of total polyphenol content and antioxidant activity in cookies formulated with different percentages of wheat flour and sunflower oil replacement using Sacha inchi cake flour and Sacha inchi oil. The results indicate that the phenolic compound content increased significantly ($p \leq 0.05$) with flour and oil substitution, reaching up to 1.46 times that of the control (formulation without substitution) in the formulation with 100% wheat flour replacement. Significant differences ($p \leq 0.05$) were observed between treatments with different levels of flour substitution; however, within the same flour level, oil substitution did not have a significant effect. These findings support previous reports indicating that Sacha inchi cake flour is a primary source of

polyphenols and they are consistent with studies showing increased polyphenol content in other baked products, such as bread, where the incorporation of Sacha inchi flour also enhanced the levels of bioactive compounds [9] Furthermore, they align with the high content of these biocomponents reported in the bromatological analysis previously conducted on this sacha inchi cake flour (**SM_Table 1 in Supplementary Materials**). This suggests that its use as a substitute for wheat flour boosts the contribution of these compounds, thereby improving the functional properties of the formulations, particularly due to its antioxidant potential and valuable nutritional profile.

From an antioxidant activity perspective, the different formulations showed promising results. According to FRAP assays, a progressive increase in antioxidant capacity of up to 1.99 times was observed as the substitution levels of both Sacha inchi flour and oil increased to 100%. Significant differences ($p \leq 0.05$) were found between formulations with different levels of flour replacement, as well as between those with varying degrees of oil substitution. This suggests that both defatted Sacha inchi cake flour and oil contributed antioxidant compounds with free radical-scavenging activity. While no direct evidence was found of polyphenol contributions from the oil, primarily from the cake flour, the increased antioxidant activity, whether due to flour or oil substitution, suggests that, in addition to the flour's effect, the oil may provide other antioxidant compounds such as carotenoids, chlorophylls, and fat-soluble vitamins. These results are consistent with findings from previous studies on other baked goods, such as bread, where the incorporation of Sacha inchi cake flour also enhanced antioxidant capacity, confirming its potential in functional food formulations [9]. These findings underline the functional potential of both Sacha inchi flour and oil, suggesting that replacing sunflower oil with Sacha inchi oil could not only provide a healthier lipid profile, but also improve the oxidative stability and antioxidant capacity of the final product. On the other hand, the results of the DPPH assay showed that the antioxidant capacity was higher in formulations with partial oil replacement compared to the control and those with Sacha inchi flour replacement. However, in formulations where both flour and oil were completely replaced, a significant decrease in antioxidant activity was observed ($p \leq 0.05$).

Overall, these results demonstrate that the partial or total replacement of wheat flour and sunflower oil with defatted Sacha inchi cake flour and Sacha inchi oil significantly influences the antioxidant activity of the cookies. Furthermore, the observed increase in polyphenols and antioxidant capacity reinforces the potential for Sacha inchi derivatives as functional ingredients. These findings highlight the potential of Sacha inchi flour and oil in baked goods, offering an alternative with antioxidant benefits and a healthier lipid profile.

Effect of incorporating Sacha inchi oil and cake flour on physical properties of cookies

As commonly acknowledged, the diameter, thickness, and spread ratio of cookies are essential indicators of dough behavior during baking and directly influence consumer perception [40]. According to the data presented in **Table 3** and **Figure 1**, the partial or complete substitution of wheat flour with Sacha inchi oil cake flour, and sunflower oil with Sacha inchi oil, had a significant impact on these physical attributes Cookie diameters ranged from 3.34 cm (50% oil cake flour and 50% sacha inchi oil) to 5.85 cm (0% oil cake flour and 100% sacha inchi oil). The control formulation (0% substitution) had a diameter of 5.41 cm, which was significantly ($p \leq 0.05$) higher than most of the formulations with Sacha inchi ingredients. Only the formulation with 100% Sacha inchi oil and no flour substitution showed a greater diameter than the control ($p \leq 0.05$), indicating that full replacement of sunflower oil with Sacha inchi oil promotes cookie spread. This observation is consistent with previous studies suggesting that oils with lower melting points enhance dough expansion during baking. In contrast, formulations containing Sacha inchi oil cake flour showed significantly reduced diameters ($p \leq 0.05$). The lowest value was observed in the formulation with 50% oil cake flour and 50% Sacha inchi oil (3.34 cm), followed by that with 100% oil cake flour and 50% Sacha inchi oil (3.58 cm) and 100% oil cake flour and 0% oil (4.64 cm). This reduction is likely due to the increased fiber and protein content, which may elevate dough viscosity and hinder spreading during baking [41,42]. The thickest cookie (1.93 cm) ($p \leq 0.05$) was observed in the formulation with 100% oil cake flour and 0% Sacha inchi oil, while the thinnest (0.90 cm) resulted from the formulation with 0% oil

cake flour and 100% Sacha inchi oil. Overall, oil-rich formulations without oil cake flour tended to produce thinner cookies, whereas those with higher oil cake flour content were thicker ($p \leq 0.05$), likely due to restricted spread and increased dough structure. The spread ratio (diameter/thickness), an integrated indicator of expansion, ranged from 2.40 (100% oil cake flour and 0% sacha inchi oil) to 6.48 (0% oil cake flour and 100% sacha inchi oil). The control (0% substitution) had a moderate spread ratio of 2.88. Notably, the formulation with 0% oil cake flour and 100% Sacha inchi oil displayed the highest spread ratio ($p \leq 0.05$), confirming sacha inchi oil's role in promoting expansion. On the other hand, combinations with 50% or 100% oil cake flour and little or no sacha inchi oil had the lowest spread ratios ($p \leq 0.05$), consistent with their higher thickness and smaller diameters. This behavior may result from the fiber and protein in the oil cake flour reducing dough extensibility and interfering with leavening agents[43]. These results confirm a clear interaction between fat and flour type in shaping cookie geometry.

Cookie color is a key sensory attribute that influences consumer acceptance. Using the CIE Lab* system, lightness (L^*), redness (a^*), and yellowness (b^*) were evaluated. Lightness values ranged from 56.76 (50% oil cake flour and 100% Sacha inchi oil) to 65.86 (0% oil cake flour and 50% Sacha inchi oil). Most formulations with Sacha inchi oil or oil cake flour showed higher L^* than the control (57.92) ($p \leq 0.05$), indicating a lighter appearance, potentially due to natural pigments and reduced Maillard browning [39]. However, full flour substitution (100% oil cake flour) did not always yield the highest lightness, suggesting complex interactions during baking. The a^* parameter (red-green axis) ranged from 0.80 (50% oil cake flour and 50% Sacha inchi oil) to 5.33 (50% oil cake flour and 100% Sacha inchi oil). Redness generally increased with higher sacha inchi oil cake flour content, possibly due to enhanced Maillard reactions from additional amino acids. Formulations containing only Sacha inchi oil had less influence on a^*, highlighting the greater impact of flour substitution ($p \leq 0.05$). Regarding b^* (yellow-blue axis), values varied from 23.52 (50% oil cake flour and 50% sacha inchi oil) to 31.67 (0% oil cake flour and 50% Sacha inchi oil). Incorporation of sacha inchi oil slightly increased b*,

while full flour substitution showed a minor decreasing effect. The formulation with 50% substitution of both oil and flour showed a high *b** value (31.19), suggesting a synergistic effect between flour and oil in enhancing yellowness. Nonetheless, some differences were not statistically significant, indicating that yellowness changes were formulation-dependent. The total color difference (ΔE) compared to the control confirmed perceptible variation across all samples. The highest ΔE was found in the formulation with 0% oil cake flour and 50% Sacha inchi oil (8.64) ($p \leq 0.05$), followed by the combination with 50% oil cake flour and 50% Sacha inchi oil (6.11), while the substitution of only 50% oil cake flour (and 0% sacha inchi oil) showed the lowest deviation (2.04). These differences likely stem from both ingredient color and chemical reactions (Maillard and caramelization) modulated by the presence of protein and fat [44].

In terms of texture, cookie hardness varied significantly. The control cookie had a hardness of 51.74 N. Substitution with 50% Sacha inchi oil (no flour replacement) increased hardness to 64.04 N, while full replacement of oil (100% Sacha inchi oil, no flour substitution) resulted in the highest hardness (128.12 N), more than 2.4 times that of the control ($p \leq 0.05$). This suggests that Sacha inchi oil leads to a denser structure, likely due to differences in fatty acid profiles and their effect on dough structure and moisture retention [45,46]. In contrast, formulations containing Sacha inchi oil cake flour showed a marked decrease in hardness ($p \leq 0.05$). Substituting 50% of the wheat flour (with no Sacha inchi oil) led to a reduction to 19.06 N, while combinations with 100% flour substitution (with or without oil) resulted in values between 8.90 N and 22.01 N, indicating a much softer texture—up to 5.8 times less firm than the control ($p \leq 0.05$). These reductions suggest that the absence of gluten and the lipid content in Sacha inchi flour contribute to a more tender structure. The combination of Sacha inchi oil and flour may also exert an emulsifying effect, softening the matrix even further, as reported in studies involving emulsifiers [47].

The results demonstrate that the type and proportion of fat and flour significantly affect cookie geometry, color, and texture. Sacha inchi oil increases spread and hardness, while Sacha inchi oil cake

flour reduces both spread and hardness, enabling targeted modulation of cookie characteristics. From a technological perspective, these ingredients offer potential for developing customized cookies with desirable texture and visual appeal, although adjustments may be required in applications where firmer textures are needed.

Conclusions

This study demonstrated that replacing wheat flour and sunflower oil with Sacha inchi derivatives significantly enhances cookies' nutritional profile and functional properties. Nutritionally, higher substitution levels increased protein, dietary fiber, omega-3 fatty acids, and essential minerals (calcium, magnesium, potassium, phosphorus, and zinc) while reducing carbohydrates and saturated fats. However, the observed decrease in iron content indicates the need for complementary iron sources in final formulations. From a functional perspective, incorporating Sacha inchi oil cake flour increased polyphenol content and antioxidant capacity, supporting its potential as an ingredient for cardiovascular and metabolic health benefits. Physical assessment revealed that Sacha inchi oil increased cookie diameter and spread, while the oil cake flour reduced diameter and increased thickness, resulting in softer cookies with full flour replacement. These changes provide opportunities for tailoring baked goods with specific sensory attributes. Overall, these findings position Sacha inchi flour and oil as promising ingredients for developing functional, nutritious, and sustainable bakery products while promoting the valorization of agro-industrial by-products and Amazonian crops in alignment with circular economy principles. Future research should address texture modifications at high substitution levels, evaluate consumer acceptance of novel flavors, establish reliable supply chains beyond small-scale farming, assess economic viability compared to conventional ingredients, and navigate regulatory requirements for novel food ingredients. Addressing these challenges through targeted research will be essential to fully realize the commercial potential of these valuable Amazonian by-products.

Declaration of Competing Interest

The authors declare that the research was conducted in the absence of any commercial or financial relationships that could be construed as a potential conflict of interest.

The author(s) declared that they were an editorial board member of Frontiers, at the time of submission. This had no impact on the peer review process and the final decision.

Acknowledgments

We would like to thank Universidad San Francisco de Quito USFQ, particularly the USFQ Research Dean's Office, for supporting Fernando E. Alejandro Ruiz and Julio F. Ortega Jácome, students of Food Engineering. We also sincerely thank the members of the Shushufindi community in Sucumbíos Province, located in the northern Amazon region of Ecuador, for their valuable collaboration in providing samples of sacha inchi seeds, oil cake, and oil. Their support and generosity were essential to the development of this study.

Funding

This work was supported by Universidad San Francisco de Quito through an Interdisciplinary Grant (Project ID: 23218) awarded to José M. Alvarez-Suárez.

References

1. Dwivedi SL, Lammerts van Bueren ET, Ceccarelli S, Grando S, Upadhyaya HD, Ortiz R. Diversifying Food Systems in the Pursuit of Sustainable Food Production and Healthy Diets. Trends Plant Sci. 2017;22: 842–856. doi:10.1016/j.tplants.2017.06.011
2. Singla D, Malik T, Singh A, Thakur S, Kumar P. Advances in understanding wheat-related disorders: A comprehensive review on gluten-free products with emphasis on wheat allergy, celiac and non-celiac gluten sensitivity. Food Chemistry Advances. 2024;4: 100627. doi:10.1016/j.focha.2024.100627
3. FAO. Crops and climate change impact briefs. Climate-smart agriculture for more sustainable, resilient, and equitable food systems. Rome: FAO; 2022. doi:10.4060/cb8030en
4. Orejuela-Escobar LM, Landázuri AC, Goodell B. Second generation biorefining in Ecuador: Circular bioeconomy, zero waste technology, environment and sustainable development: The nexus. Journal of Bioresources and Bioproducts. 2021;6: 83–107. doi:10.1016/j.jobab.2021.01.004
5. Ronie ME, Abdul Aziz AH, Kobun R, Pindi W, Roslan J, Putra NR, et al. Unveiling the potential applications of plant by-products in food – A review. Waste Management Bulletin. 2024;2: 183–203. doi:10.1016/j.wmb.2024.07.008

Commented [T5]: Please write all references according to Journal guidelines in Vancouver style, mistakes like non abbreviated journals names, space issues etc are present, deeply attention is required in this regards.

6. Hoehnel A, Zannini E, Arendt EK. Targeted formulation of plant-based protein-foods: Supporting the food system's transformation in the context of human health, environmental sustainability and consumer trends. Trends Food Sci Technol. 2022;128: 238–252. doi:10.1016/j.tifs.2022.08.007
7. del-Castillo ÁMR, Gonzalez-Aspajo G, de Fátima Sánchez-Márquez M, Kodahl N. Ethnobotanical Knowledge in the Peruvian Amazon of the Neglected and Underutilized Crop Sacha Inchi (Plukenetia volubilis L.). Econ Bot. 2019;73: 281–287. doi:10.1007/s12231-019-09459-y
8. Narváez Cadena RA, Salas Zambrano AP, Bravo Gómez JE, Muñoz Pabon KS, Roa-Acosta DF. Characterization of sacha inchi (Plukenetia volubilis) and taro (Colocasia esculenta) flours with potential application in the preparation of both gluten-free and high protein foods. F1000Res. 2025;12: 378. doi:10.12688/f1000research.130394.4
9. Martínez E, Ramos-Escudero F. Valorization of flours from cocoa, sinami and sacha inchi by-products for the reformulation of Peruvian traditional flatbread ('Pan Chapla'). Int J Gastron Food Sci. 2024;36: 100930. doi:10.1016/j.ijgfs.2024.100930
10. Landines Vera E, Villacrés E, Coello Ojeda K, Guadalupe Moyano V, Quezada Tobar M, Quelal MB, et al. Evaluation of antinutrients, nutritional, and functional properties in sacha inchi (Plukenetia volubilis L) cake treated with hydrothermal processes. Heliyon. 2024;10: e37291. doi:10.1016/j.heliyon.2024.e37291
11. Gonzales GF, Gonzales C. A randomized, double-blind placebo-controlled study on acceptability, safety and efficacy of oral administration of sacha inchi oil (Plukenetia volubilis L.) in adult human subjects. Food and Chemical Toxicology. 2014;65: 168–176. doi:10.1016/j.fct.2013.12.039
12. Auquiñivin Silva EA, Castro Alayo EM. Elaboración de galletas enriquecidas a partir de una mezcla de cereales, leguminosas y tubérculos. Chachapoyas, región Amazonas. Industrial Data. 2015;18: 84–90.
13. AOAC. AOAC Official Method: 925.10-1925 Solids (total) and loss on drying (moisture) in flour. Air oven method. 19th ed. In: George Latimer Jr., editor. Official Methods of Analysis of AOAC International. 19th ed. Gaithersburg; 2012.
14. AOAC. AOAC Official Method: 923.03 Ash of flour. Direct method. 15th ed. In: Kenneth Helrich, editor. Official Methods of Analysis of AOAC International. 15th ed. Gaithersburg: Association of Official Analytical Chemists.; 1990.
15. AOAC. AOAC Official Method 978.10: Fiber (crude) in animal feed and pet food. Fritted glass crucible method. 16th ed. Official Methods of Analysis of AOAC International. 16th ed. Washington D.C., USA.: Association of Official Analytical Chemists.; 1996.
16. AACC. Total Dietary Fiber. 11th ed. AACC International Approved Methods. 11th ed. St. Paul, MN, U.S.A.: AACC International; 2009. doi:10.1094/AACCIntMethod-32-05.01
17. AOAC. AOAC Official Method:985.29: Total Dietary Fiber in Foods. 17th ed. Official Methods of Analysis of AOAC International. 17th ed. Gaithersburg: Association of Official Analytical Chemists.; 2003.
18. AOAC. AOAC Official Method: 920.39: Fat (crude) or ether extract in animal feed. 15th ed. In: Kenneth Helrich, editor. Official Methods of Analysis of AOAC International. 15th ed. Gaithersburg: Association of Official Analytical Chemists.; 1990.
19. AOAC. AOAC Official Method: 2001.11 Protein (crude) in animal feed,Forage (plant tissue),Grain,and oilseeds. Block digestion method using copper catalyst and steam distillation into boric acid. 18th ed. In: William Horwitz, editor. Official Methods of Analysis of AOAC International. 18th ed. Gaithersburg: Association of Official Analytical Chemists.; 2005.

20. Plustea L, Negrea M, Cocan I, Radulov I, Tulcan C, Berbecea A, et al. Lupin (Lupinus spp.)-Fortified Bread: A Sustainable, Nutritionally, Functionally, and Technologically Valuable Solution for Bakery. Foods. 2022;11: 2067. doi:10.3390/foods11142067
21. AC Analytical Controls. EN 14103 Determination of total FAME and linolenic acid methyl ester in FAME with AC Biodiesel All in One Solution. 2012. Available: https://www.paclp.com/tenants/pac/documents/ac_en14103_totalfame_biodiesel.pdf
22. Ponce EL, Ochoa-Herrera V, Quintanilla F, Egas DA, Mora JR. Optimization of a Gas Chromatography Methodology for Biodiesel Analysis. Journal of Analytical Chemistry. 2021;76: 106–111. doi:10.1134/S1061934821010093
23. AOAC. AOAC Official Method: 2015.01.Heavy Metals in Food. 18th ed. Official Methods of Analysis of AOAC International. 18th ed. Gaithersburg: Association of Official Analytical Chemists.; 2015.
24. Sokmen O, Ozdemir S, Dundar AN, Cinar A. Quality properties and bioactive compounds of reduced-fat cookies with bee pollen. Int J Gastron Food Sci. 2022;29: 100557. doi:10.1016/j.ijgfs.2022.100557
25. Singleton VL, Orthofer R, Lamuela-Raventós RM. Analysis of total phenols and other oxidation substrates and antioxidants by means of folin-ciocalteu reagent. Methods in Enzymology. 1999. pp. 152–178. doi:10.1016/S0076-6879(99)99017-1
26. Benzie IFF, Strain JJ. The ferric reducing ability of plasma (FRAP) as a measure of “antioxidant power”: The FRAP assay. Anal Biochem. 1996;239: 70–76. doi:10.1006/abio.1996.0292
27. Prymont-Przyminska A, Zwolinska A, Sarniak A, Wlodarczyk A, Krol M, Nowak M, et al. Consumption of strawberries on a daily basis increases the non-urate 2,2-diphenyl-1-picryl-hydrazyl (DPPH) radical scavenging activity of fasting plasma in healthy subjects. J Clin Biochem Nutr. 2014;55: 48–55. doi:10.3164/jcbn.13-93
28. AACC. Approved Methods of the AACC. 10th ed. St. Paul.: American Association of Cereal Chemists; 2000.
29. Korese JK, Chikpah SK, Hensel O, Pawelzik E, Sturm B. Effect of orange-fleshed sweet potato flour particle size and degree of wheat flour substitution on physical, nutritional, textural and sensory properties of cookies. European Food Research and Technology. 2021;247: 889–905. doi:10.1007/s00217-020-03672-z
30. Sahin S, Gülüm Sumnu S. Physical Properties of Foods. New York, NY: Springer New York; 2006. doi:10.1007/0-387-30808-3
31. Belitz H-D, Grosch W, Peter Schieberle P. Food Chemistry. 4 th. Springer Science & Business Media, editor. Berlin, Heidelberg: Springer Berlin Heidelberg; 2009. doi:10.1007/978-3-540-69934-7
32. Torres Sánchez EG, Hernández-Ledesma B, Gutiérrez L-F. Sacha Inchi Oil Press-cake: Physicochemical Characteristics, Food-related Applications and Biological Activity. Food Reviews International. 2023;39: 148–159. doi:10.1080/87559129.2021.1900231
33. Wang S, Zhu F, Kakuda Y. Sacha inchi (Plukenetia volubilis L.): Nutritional composition, biological activity, and uses. Food Chem. 2018;265: 316–328. doi:10.1016/j.foodchem.2018.05.055
34. Khorasaniha R, Olof H, Voisin A, Armstrong K, Wine E, Vasanthan T, et al. Diversity of fibers in common foods: Key to advancing dietary research. Food Hydrocoll. 2023;139: 108495. doi:10.1016/j.foodhyd.2023.108495
35. McRorie JW, McKeown NM. Understanding the Physics of Functional Fibers in the Gastrointestinal Tract: An Evidence-Based Approach to Resolving Enduring Misconceptions about Insoluble and Soluble Fiber. J Acad Nutr Diet. 2017;117: 251–264. doi:10.1016/j.jand.2016.09.021

36. Salleh SN, Fairus AAH, Zahary MN, Bhaskar Raj N, Mhd Jalil AM. Unravelling the Effects of Soluble Dietary Fibre Supplementation on Energy Intake and Perceived Satiety in Healthy Adults: Evidence from Systematic Review and Meta-Analysis of Randomised-Controlled Trials. Foods. 2019;8: 15. doi:10.3390/foods8010015
37. Alahmari LA. Dietary fiber influence on overall health, with an emphasis on CVD, diabetes, obesity, colon cancer, and inflammation. Front Nutr. 2024;11. doi:10.3389/fnut.2024.1510564
38. USDA. Oil, sunflower. FoodData Central. 2021 [cited 19 Feb 2025]. Available: https://fdc.nal.usda.gov/food-details/1750349/nutrients
39. Khan SU, Lone AN, Khan MS, Virani SS, Blumenthal RS, Nasir K, et al. Effect of omega-3 fatty acids on cardiovascular outcomes: A systematic review and meta-analysis. EClinicalMedicine. 2021;38: 100997. doi:10.1016/j.eclinm.2021.100997
40. Owheruo JO, Edo GI, Makia RS, Gaaz TS, Okolie MC, Nwaogu MU, et al. Nutritional qualities of cookies made from wheat/cashew nut composite flour. Food and Humanity. 2024;3: 100452. doi:10.1016/j.foohum.2024.100452
41. Jacob J, Leelavathi K. Effect of fat-type on cookie dough and cookie quality. J Food Eng. 2007;79: 299–305. doi:10.1016/j.jfoodeng.2006.01.058
42. Pareyt B, Delcour JA. The Role of Wheat Flour Constituents, Sugar, and Fat in Low Moisture Cereal Based Products: A Review on Sugar-Snap Cookies. Crit Rev Food Sci Nutr. 2008;48: 824–839. doi:10.1080/10408390701719223
43. Jemziya MBF, Mahendran T. Physical quality characters of cookies produced from composite blends of wheat and sweet potato flour. Ruhuna Journal of Science. 2017;8: 12. doi:10.4038/rjs.v8i1.23
44. Olukomaiya OO, Adiamo OQ, Fernando WC, Mereddy R, Li X, Sultanbawa Y. Effect of solid-state fermentation on proximate composition, anti-nutritional factor, microbiological and functional properties of lupin flour. Food Chem. 2020;315: 126238. doi:10.1016/j.foodchem.2020.126238
45. Seker IT, Ozboy-Ozbas O, Gokbulut I, Ozturk S, Koksel H. Utilization of apricot kernel flour as fat replacer in cookies. J Food Process Preserv. 2010;34: 15–26. doi:10.1111/j.1745-4549.2008.00258.x
46. Gutiérrez-Luna K, Astiasaran I, Ansorena D. Fat reduced cookies using an olive oil-alginate gelled emulsion: Sensory properties, storage stability and in vitro digestion. Food Research International. 2023;167: 112714. doi:10.1016/j.foodres.2023.112714
47. Ashwath Kumar K, Sudha ML. Effect of fat and sugar replacement on rheological, textural and nutritional characteristics of multigrain cookies. J Food Sci Technol. 2021;58: 2630–2640. doi:10.1007/s13197-020-04769-9

FIGURE CAPTION

Figure 1. Sides and tops appearance of cookies formulations

Ctl: 0% sacha inchi oil cake flour, 0% sacha inchi oil; **F1:** 0% oil cake flour, 50% sacha inchi oil; **F2:** 0% oil cake flour, 100% sacha inchi oil; **F3:** 50% oil cake flour, 0% sacha inchi oil; **F4:** 50% oil cake flour, 50% sacha inchi oil; **F5:** 50% oil cake flour, 100% sacha inchi oil; **F6:** 100% oil cake flour, 0%

sacha inchi oil; **F7:** 100% oil cake flour, 50% sacha inchi oil; **F8:** 100% oil cake flour, 100% sacha inchi oil.

Table 1. Recipe for cookie formulation

Ingredients (%)	Ctl	F1	F2	F3	F4	F5	F6	F7	F8
Wheat flour	100	100	100	50	50	50	0	0	0
Sacha inchi oil cake flour	0	0	0	50	50	50	100	100	100
Sacha inchi oil	0	33.33	66.67	0	33.33	66.67	0	33.33	66.67
Sunflower oil	66.67	33.33	0	66.67	33.33	0	66.67	33.33	0
Egg	27.78	27.78	27.78	27.78	27.78	27.78	27.78	27.78	27.78
Vanilla flavor	1	1	1	1	1	1	1	1	1
Sodium bicarbonate	1.39	1.39	1.39	1.39	1.39	1.39	1.39	1.39	1.39
Sucrose (fine granulated)	27.78	27.78	27.78	27.78	27.78	27.78	27.78	27.78	27.78
Brownulated granulated sucrose	55.56	55.56	55.56	55.56	55.56	55.56	55.56	55.56	55.56
Honey	2.33	2.33	2.33	2.33	2.33	2.33	2.33	2.33	2.33
Salt	1.39	1.39	1.39	1.39	1.39	1.39	1.39	1.39	1.39

Ctl: 0% sacha inchi oil cake flour, 0% sacha inchi oil; **F1:** 0% oil cake flour, 50% sacha inchi oil; **F2:** 0% oil cake flour, 100% sacha inchi oil; **F3:** 50% oil cake flour, 0% sacha inchi oil; **F4:** 50% oil cake flour, 50% sacha inchi oil; **F5:** 50% oil cake flour, 100% sacha inchi oil; **F6:** 100% oil cake flour, 0% sacha inchi oil; **F7:** 100% oil cake flour, 50% sacha inchi oil; **F8:** 100% oil cake flour, 100% sacha inchi oil.

Table 2. Proximal composition and chemical composition of control and cookies with Sacha inchi oil cake flour and oil substitutes

Analysis	Cookie's formulations								
	Ctl	F1	F2	F3	F4	F5	F6	F7	F8
Proximate									
Moisture (%)	2.22±0.01^{a}	2.21±0.03^{a}	2.22±0.03^{a}	2.38±0.01^{b}	2.35±0.03^{b}	2.36±0.03^{b}	2.54±0.03^{c}	2.55±0.04^{c}	2.57±0.07^{c}
Water activity (a_w)	0.52±0.03^{a}	0.54±0.02^{a}	0.52±0.05^{a}	0.39±0.02^{b}	0.41±0.01^{b}	0.42±0.03^{b}	0.26±0.01^{c}	0.25±0.02^{c}	0.25±0.01^{c}
Ash (%)	1.05±0.05^{a}	1.06±0.05^{a}	1.07±0.02^{a}	1.61±0.01^{b}	1.65±0.03^{b}	1.70±0.02^{b}	2.39±0.02^{c}	2.34±0.05^{c}	2.38±0.07^{c}
Protein (%)	8.83±0.22^{a}	8.09±0.72^{a}	7.47±1.23^{a}	15.18±0.31^{b}	14.54±0.25^{b}	14.19±0.39^{b}	19.45±0.57^{c}	19.49±1.14^{c}	19.34±0.50^{c}
Fat (%)	19.18±0.68^{a}	22.86±0.44^{a}	22.77±1.82^{a}	27.70±0.93^{b}	28.89±1.09^{b}	28.70±0.46^{b}	31.60±0.87^{c}	34.68±0.36^{c}	32.77±1.82^{c}
Crude fiber (%)	0.55±0.02^{a}	0.55±0.05^{a}	0.57±0.04^{a}	1.81±0.12^{b}	1.84±0.05^{b}	1.80±0.08^{b}	3.22±0.17^{c}	3.29±0.11^{c}	3.25±0.10^{c}
Total carbohydrate (%)	68.72±0.45^{a}	65.77±0.32^{a}	66.47±1.90^{a}	54.19±1.23^{b}	52.56±1.02^{b}	53.05±0.69^{b}	43.93±0.43^{c}	40.93±0.85^{c}	42.93±1.81^{c}
Energy value (kcal/100 g)	482.82±3.54^{a}	501.10±2.24^{b}	500.71±6.43^{b}	517.54±4.66^{c}	528.45±5.34^{c}	527.28±2.31^{c}	538.72±4.35^{d}	553.84±1.74^{e}	544.06±9.62^{d}
Dietary fiber (%)									
Soluble fiber (%)	1.34±0.25	1.55±0.12	1.35±0.23	0.95±0.13	1.40±0.23	2.52±0.80	1.67±0.23	0.75±0.10	2.02±0.28
Insoluble fiber (%)	1.02±0.04	1.03±0.43	1.05±0.04	6.36±0.34	8.31±0.45	7.76±0.32	12.98±0.60	22.25±1.41	71.59±0.87
Fatty acids composition (g/100 g)									
Palmitic acid (C16)	2.10±0.61^{a}	1.86±0.79^{b}	1.91±0.90^{b}	0.92±0.45^{c}	1.88±0.82^{b}	1.62±0.10^{b}	1.73±0.48^{b}	1.69±0.46^{b}	1.72±0.65^{b}
Stearic acid (C18)	1.28±0.34^{a}	1.23±0.53^{a}	1.27±0.16^{a}	0.57±0.02^{b}	1.30±0.15^{a}	2.41±0.12^{c}	1.84±0.11^{d}	1.12±0.19^{e}	1.13±0.23^{e}
Oleic acid (C18)	12.78±2.33^{a}	8.33±1.82^{b}	5.87±0.57^{c}	6.76±0.96^{d}	6.12±0.52^{d}	6.44±0.85^{d}	5.83±0.69^{a}	5.56±0.23^{a}	3.43±0.86^{e}
Linoleic acid (C18)	12.93±1.87^{a}	10.46±1.78^{b}	12.51±1.68^{a}	9.89±0.53^{c}	10.14±1.58^{c}	10.90±1.86^{c}	9.74±1.07^{c}	9.75±0.40^{c}	11.53±1.26^{b}
Linolenic acid (C18)	2.55±0.11^{a}	4.89±0.23^{b}	7.23±1.13^{c}	2.59±0.24^{a}	6.23±0.77^{c}	9.34±1.17^{d}	4.32±0.99^{b}	6.63±0.02^{c}	7.88±1.91^{c}
Macro and microelements (mg/100 g)									
Macroelements									
Calcium (Ca)	15.19±0.16^{a}	14.86±0.76^{a}	15.11±0.50^{a}	80.76±1.54^{b}	76.84±4.21^{b}	78.01±1.23^{b}	157.13±9.08^{c}	159.36±5.18^{c}	164.22±3.20^{c}
Magnesium (Mg)	15.47±0.48^{a}	14.30±0.25^{a}	14.90±0.19^{a}	75.63±1.49^{b}	73.03±1.01^{b}	75.40±1.73^{b}	152.19±9.03^{c}	154.23±3.62^{c}	157.26±3.83^{c}
Potassium (K)	83.59±3.64^{a}	81.61±1.79^{a}	80.16±2.58^{a}	180.43±3.24^{b}	177.13±5.64^{b}	179.72±4.09^{b}	234.21±4.39^{c}	236.68±8.13^{c}	240.98±12.67^{c}
Sodium (Na)	308.63±2.37^{a}	312.55±9.87^{a}	310.43±4.36^{a}	308.74±14.46^{a}	305.93±5.73^{a}	311.39±7.19^{a}	306.61±12.59^{a}	307.19±5.51^{a}	313.62±9.94^{a}
Phosphorus (P)	75.24±3.03^{a}	72.53±2.88^{a}	73.57±3.64^{a}	238.21±3.94^{b}	246.48±3.41^{b}	239.54±4.27^{b}	399.59±6.87^{b}	401.10±11.13^{b}	404.36±14.45^{b}
Microelements									
Iron (Fe)	2.10±0.05^{a}	2.14±0.01^{a}	2.11±0.22^{a}	1.58±0.09^{b}	1.64±0.14^{b}	1.65±0.03^{b}	1.55±0.09^{b}	1.49±0.07^{b}	1.56±0.12^{b}
Zinc (Zn)	0.57±0.02^{a}	0.52±0.01^{a}	0.50±0.01^{a}	1.76±0.02^{b}	1.66±0.02^{b}	1.69±0.01^{b}	2.64±0.04^{c}	2.57±0.11^{c}	2.61±0.44^{c}
Bioactivity									

TPC (mg GAEq/100 g)	4.79±0.13^{a}	4.90±0.12^{a}	5.05±0.10^{a}	6.53±0.35^{b}	6.85±0.03^{b}	6.90±0.07^{c}	6.76±0.06^{c}	6.89±0.05^{c}	6.99±0.07^{c}
FRAP assay (mM TEq/g FW)	51.05±1.29^{a}	71.55±3.5^{b}	85.04±4.15^{c}	55.45±1.92^{a}	66.19±1.41^{d}	78.57±2.55^{e}	70.89±2.78^{b}	78.55±2.78^{f}	101.55±5.40^{g}
DPPH assay (mM TEq/g FW)	21.12±0.99^{a}	24.32±1.50^{b}	23.42±1.53^{b}	18.67±0.70^{c}	20.23±1.38^{a}	20.04±1.14^{a}	16.99±1.20^{d}	17.57±1.19^{d}	17.17±2.30^{d}

Data represent the mean ± standard deviation. Different superscript letters indicate significantly different mean values ($p < 0.05$).

Ctl: 0% sacha inchi oil cake flour, 0% sacha inchi oil; **F1:** 0% oil cake flour, 50% sacha inchi oil; **F2:** 0% oil cake flour, 100% sacha inchi oil; **F3:** 50% oil cake flour, 0% sacha inchi oil; **F4:** 50% oil cake flour, 50% sacha inchi oil; **F5:** 50% oil cake flour, 100% sacha inchi oil; **F6:** 100% oil cake flour, 0% sacha inchi oil; **F7:** 100% oil cake flour, 50% sacha inchi oil; **F8:** 100% oil cake flour, 100% sacha inchi oil.

Table 3. Dimensions, color parameters and hardness of the cookie's formulations

Formulation	Diameter (cm)	Thickness (cm)	Spread factor	*L**	*a**	*b**	ΔE	Hardness (N)
Ctl	5.41±0.02^{c}	1.88±0.02^{b}	2.88±0.02^{e}	57.92±1.09^{de}	1.91±0.06^{c}	28.93±0.25^{bc}	--	51.74±6.16^{bc}
F1	4.60±0.07^{e}	1.24±0.02^{e}	3.70±0.03^{d}	65.86±0.65^{a}	1.52±0.76^{c}	31.67±1.83^{a}	8.64±1.13^{a}	64.04±7.38^{b}
F2	5.85±0.08^{a}	0.90±0.03^{f}	6.48±0.14^{a}	61.23±1.44^{bc}	1.58±0.15^{c}	29.30±0.54^{bc}	3.37±0.33^{de}	128.12±6.89^{a}
F3	5.68±0.09^{b}	1.44±0.03^{d}	3.94±0.10^{c}	59.41±1.53^{cde}	2.96±0.37^{b}	30.47±0.16^{ab}	2.04±0.04^{f}	19.06±4.13^{d}
F4	3.34±0.05^{g}	1.27±0.04^{e}	2.64±0.12^{f}	58.60±2.06^{cde}	0.80±0.02^{d}	23.52±0.77^{d}	6.11±0.66^{b}	19.95±6.90^{d}
F5	5.03±0.06^{d}	0.91±0.05^{f}	5.49±0.22^{b}	56.76±3.47^{e}	5.33±0.43^{a}	31.19±2.10^{a}	5.44±1.17^{bc}	32.49±7.56^{cd}
F6	4.64±0.02^{e}	1.93±0.04^{a}	2.40±0.06^{g}	63.08±1.55^{ab}	3.03±0.24^{b}	28.49±0.23^{c}	4.48±0.34^{cd}	22.01±3.32^{cd}
F7	3.58±0.04^{f}	1.25±0.02^{e}	2.87±0.05^{e}	60.08±0.55^{cd}	2.12±0.57^{c}	27.83±0.63^{c}	3.19±0.81^{ef}	8.90±2.07^{d}
F8	4.59+0.07^{e}	1.55+0.04^{c}	2.96+0.07^{e}	60.64+1.05^{bcd}	1.99+0.39^{c}	27.79+0.60^{c}	3.03+0.42^{ef}	8.94+2.70^{d}

Data represent the mean ± standard deviation. Different superscript letters indicate significantly different mean values ($p < 0.05$).

Ctl: 0% sacha inchi oil cake flour, 0% sacha inchi oil; **F1:** 0% oil cake flour, 50% sacha inchi oil; **F2:** 0% oil cake flour, 100% sacha inchi oil; **F3:** 50% oil cake flour, 0% sacha inchi oil; **F4:** 50% oil cake flour, 50% sacha inchi oil; **F5:** 50% oil cake flour, 100% sacha inchi oil; **F6:** 100% oil cake flour, 0% sacha inchi oil; **F7:** 100% oil cake flour, 50% sacha inchi oil; **F8:** 100% oil cake flour, 100% sacha inchi oil

Figure 1

Click here to access/download;Figure;Alvarez-Suarez_et_al_PLOS_Fig_1.pdf

FIGURE 1

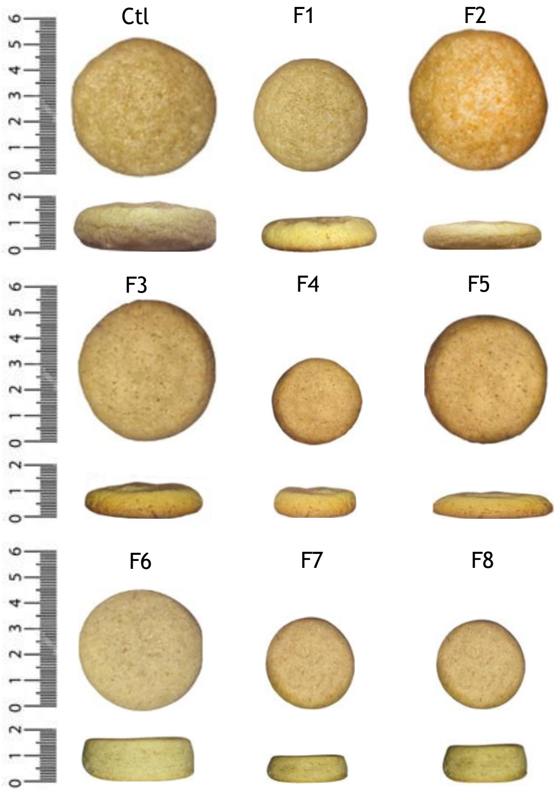

Click here to access/download
Supporting Information
Alvarez-Suarez_et_al_S_Materials.pdf
