# Supplementary material for: Influence of partial or total substitution of wheat flour and sunflower oil with Sacha inchi (Plukenetia volubilis L.) flour and oil on the quality and nutritional properties of cookies
Source: PLoS One. 2026 Jan 6;21(1):e0340190. doi: 10.1371/journal.pone.0340190 (PMC12774371; doi:10.1371/journal.pone.0340190)
Supplement: Attachment — Submitted filename: RESPONSE TO REVIEWERS.pdf [file pone.0340190.s004.pdf]

**Journal Requirements:**

When submitting your revision, we need you to address these additional requirements.

1. Please ensure that your manuscript meets PLOS ONE's style requirements, including those for file naming. The PLOS ONE style templates can be found at

https://journals.plos.org/plosone/s/file?id=wjVg/PLOSOne_formatting_sample_main_body.pdf and

https://journals.plos.org/plosone/s/file?id=ba62/PLOSOne_formatting_sample_title_authors_affiliations.pdf

**RESPONSE:** We confirm that the manuscript has been revised to fully comply with PLOS ONE's style and formatting requirements. All elements—including structure, section headings, file naming conventions, and manuscript layout—have been carefully adapted according to the official templates and author guidelines provided on the journal's website. We have ensured that the revised submission adheres to the formatting standards indicated in the links supplied by the Editorial Office.

2. Please note that funding information should not appear in any section or other areas of your manuscript. We will only publish funding information present in the Funding Statement section of the online submission form. Please remove any funding-related text from the manuscript.

**RESPONSE:** We thank the Editorial Office for the clarification. Following your instructions, we have carefully reviewed the entire manuscript and removed all funding-related information from the text. The funding details now appear exclusively in the Funding Statement section of the online submission form, as requested.

3. Thank you for stating the following financial disclosure:

"This work was supported by Universidad San Francisco de Quito through an Interdisciplinary Grant (Project ID: 23218) awarded to José M. Alvarez-Suárez."

Please state what role the funders took in the study. If the funders had no role, please state: "The funders had no role in study design, data collection and analysis, decision to publish, or preparation of the manuscript."

If this statement is not correct you must amend it as needed.

Please include this amended Role of Funder statement in your cover letter; we will change the online submission form on your behalf.

RESPONSE: The statement "The funders had no role in study design, data collection and analysis, decision to publish, or preparation of the manuscript." was included in the cover letter

4. We note that your Data Availability Statement is currently as follows: All relevant data are within the manuscript and in Supporting Information files.

Please confirm at this time whether or not your submission contains all raw data required to replicate the results of your study. Authors must share the "minimal data set" for their submission. PLOS defines the minimal data set to consist of the data required to replicate all study findings reported in

the article, as well as related metadata and methods (https://journals.plos.org/plosone/s/data-availability#loc-minimal-data-set-definition).

For example, authors should submit the following data:

- The values behind the means, standard deviations and other measures reported;

- The values used to build graphs;

- The points extracted from images for analysis.

Authors do not need to submit their entire data set if only a portion of the data was used in the reported study.

If your submission does not contain these data, please either upload them as Supporting Information files or deposit them to a stable, public repository and provide us with the relevant URLs, DOIs, or accession numbers. For a list of recommended repositories, please see https://journals.plos.org/plosone/s/recommended-repositories.

If there are ethical or legal restrictions on sharing a de-identified data set, please explain them in detail (e.g., data contain potentially sensitive information, data are owned by a third-party organization, etc.) and who has imposed them (e.g., an ethics committee). Please also provide contact information for a data access committee, ethics committee, or other institutional body to which data requests may be sent. If data are owned by a third party, please indicate how others may request data access.

**RESPONSE:** We confirm that the submission provides all the statistically processed data required to replicate the results presented in the study. Although raw, unprocessed instrumental outputs are not included, all values used for the statistical analyses—such as means, standard deviations, and the numerical data underlying the figures and tables

5. Please include captions for your Supporting Information files at the end of your manuscript, and update any in-text citations to match accordingly. Please see our Supporting Information guidelines for more information: http://journals.plos.org/plosone/s/supporting-information.

**RESPONSE:** We appreciate the comment. However, this point is no longer applicable. The data originally included as supplementary information were published separately. Therefore, in the revised version, we have removed these supplementary files and now refer directly to the published article where these results are available. Consequently, the titles of these files are no longer relevant, and the in-text citations have been updated to reflect the formal publication of these data.

**Reviewer #1:** Based on a critical review of the manuscript titled "Influence of partial or total substitution of wheat flour and sunflower oil with Sacha inchi (Plukenetia volubilis L.) flour and oil on the quality and nutritional properties of cookies", here are detailed comments section-wise, including line and page numbers

Line 39: The phrase “reaching 19.65%, more than double the control” is impactful but would benefit from a statistical reference (e.g., p-value).

Line 43: The decrease in iron content by 25% is mentioned, but the nutritional implications of this reduction are not discussed.

Line 45: “According with the lipid profiling” should be corrected to “according to the lipid profiling.”

Line 49: The contrast between Sacha inchi flour reducing hardness and oil increasing it is interesting—consider briefly explaining the mechanism.

Line 53: The phrase “suggesting interactions between lipid, protein, and fiber components” is vague—consider specifying the nature of these interactions.

Line 56: The phrase “valorization of underutilized Amazonian crops” is strong—consider briefly mentioning sustainability or economic impact.

**RESPONSE:** We thank the reviewer for their thoughtful comments. In response to Reviewer 3’s recommendations, the Abstract has been revised to comply with the PLOS ONE format (Background, Methodology, Results, and Conclusions). As a result of this restructuring, the specific phrases referred to by the reviewer have been removed and are therefore no longer present in the revised version of the Abstract. Furthermore, since the Abstract must follow a defined structure and a limited word count, the additional details suggested by the reviewer (e.g., statistical references, mechanistic explanations, and nutritional or sustainability implications) will be appropriately addressed in the Results and Discussion section of the manuscript, where they can be presented with sufficient depth and context.

Line 71: The phrase “monoculture-based grain production” could benefit from a citation or example.

**RESPONSE:** We thank the reviewer for this helpful observation. Following this suggestion, an example and a supporting reference have been added to clarify and strengthen the statement. The revised sentence now reads as follows:

> The baking industry, which has traditionally relied on refined wheat flour and conventional vegetable oils, now faces multiple challenges, including the prevalence of gluten intolerance, rising cases of metabolic disorders [2], and the environmental impact of monoculture-based grain production, such as intensive wheat and maize cultivation, which contributes to soil degradation, biodiversity loss, and increased greenhouse gas emissions [3].


- FAO. The State of the World’s Biodiversity for Food and Agriculture. Rome; 2020.

Line 93: “Particularly recognized for its remarkable protein and lipid profile” is a strong claim—consider referencing specific studies.

**RESPONSE:** We thank the reviewer for this valuable observation. To substantiate this statement, we have included two supporting references that highlight both the growing scientific attention and commercial relevance of Sacha inchi due to its nutritional composition, bioactive potential, and applications in food and health industries. The revised sentence is now properly supported by the following references:

- Wang S, Zhu F, Kakuda Y. Sacha inchi (Plukenetia volubilis L.): Nutritional composition, biological activity, and uses. Food Chemistry. 2018;265: 316–328. doi:10.1016/j.foodchem.2018.05.055
- Kodahl N. Sacha inchi (Plukenetia volubilis L.)—from lost crop of the Incas to part of the solution to global challenges? Planta. 2020;251: 80. doi:10.1007/s00425-020-03377-3

Line 98: The term “underutilized by-product” is appropriate, but the potential safety concerns of using oil cake in food should be acknowledged.

**RESPONSE:** We thank the reviewer for this important observation. Following this valuable suggestion, we have included a clarification in the text to acknowledge the potential safety concerns related to the presence of antinutritional compounds in the raw oil cake and to highlight that these can be effectively reduced through appropriate processing. The revised text now reads as follows:

> Nevertheless, despite its promising nutritional composition, the raw oil cake may contain certain antinutritional compounds, including alkaloids, oxalates, nitrates, tannins, thiocyanates, saponins, phytic acid, trypsin inhibitors, and glucosinolates, which can affect protein digestibility and mineral bioavailability if not properly processed. These compounds are, however, largely reduced through technofunctional processing steps such as roasting, drying, extrusion, or baking, as well as hydrothermal treatments (autoclaving or hot air), which have been shown to significantly decrease their levels in Sacha inchi oil cake [11].

- Landines Vera E, Villacrés E, Coello Ojeda K, Guadalupe Moyano V, Quezada Tobar M, Quelal MB, et al. Evaluation of antinutrients, nutritional, and functional properties in sacha inchi (*Plukenetia volubilis* L) cake treated with hydrothermal processes. Heliyon. 2024;10: e37291. doi:10.1016/j.heliyon.2024.e37291

Line 106: “Inconsistent extraction efficiency” is mentioned—quantitative data or references would strengthen this point.

**RESPONSE:** We thank the reviewer for this helpful comment. Following their suggestion, a supporting reference has been added to provide quantitative context and substantiate the statement regarding extraction efficiency variability. The revised sentence now reads as follows:

> Despite the promising nutritional profile of Sacha inchi, several practical challenges limit the commercial utilization of its by-products, including variable extraction efficiencies ranging from 70% to 90% depending on seed origin and processing conditions [15], underdeveloped supply chains, and potential cost disadvantages compared to conventional ingredients.

- Vásquez-Ocmín PG, Freitas Alvarado L, Sotero Solís V, Paván Torres R, Mancini-Filho J. Chemical characterization and oxidative stability of the oils from three morphotypes of Mauritia flexuosa L.f, from the Peruvian Amazon. Grasas y Aceites. 2010;61: 390–397. doi:10.3989/gya.010110.

Line 111: "Significant gap in understanding" is a strong claim—consider citing a recent review or meta-analysis to support it.

**RESPONSE:** We thank the reviewer for this constructive comment. To substantiate this statement, we have added a recent review by Goyal et al. (2022), which provides comprehensive evidence that current Sacha inchi research is largely focused on oil composition and extraction parameters, while limited attention has been given to studies evaluating the simultaneous use of Sacha inchi oil and flour in food formulations. The revised sentence now reads as follows:

> While interest in Sacha inchi as a functional ingredient is growing, research examining the simultaneous application of both its flour and oil as direct replacements for traditional baking ingredients remains limited, creating a significant gap in understanding their combined effects in everyday food products. Recent reviews have highlighted that most studies on Sacha inchi focus primarily on the compositional and physicochemical properties of the oil, with comparatively scarce investigations addressing the integration of both fractions—oil and flour—within food matrices or processing systems [16]. This lack of integrated research constrains a comprehensive understanding of the functional and technological synergies between lipid and protein components derived from this underutilized Amazonian crop**.**

Line 119: The phrase "cardioprotective benefits" should be supported by a reference or clarified as a hypothesis.

**RESPONSE:** We thank the reviewer for this accurate observation. In response, the sentence has been revised to avoid any possible misunderstanding, as the study did not directly evaluate cardioprotective effects. Instead, our objective was to develop formulations with a health-beneficial fatty acid profile, characteristic of Sacha inchi oil. Since this section of the manuscript outlines the study's objectives, we consider it inappropriate to include bibliographic references at this point. Therefore, a general descriptive term has been used, which clearly conveys the intended meaning without the need for citation. The revised sentence now reads as follows:

> Additionally, we analyzed fatty acid profiles to obtain formulations with a health-beneficial lipid composition, characteristic of Sacha inchi oil, and measured polyphenol content and antioxidant capacity to quantify the functional properties these Amazonian ingredients contribute to the final products.

Line 149 (Page 14): Table 1 is referenced but not discussed in detail—consider summarizing key differences in formulations.

**RESPONSE:** We thank the reviewer for this helpful observation. In response, a brief summary of the main formulation differences has been added to the text to provide clearer context for Table 1. The revised paragraph now highlights the three substitution gradients applied—(i) partial and total replacement of sunflower oil with Sacha inchi oil, (ii) 50 % replacement of wheat flour with oilcake flour combined with variable oil levels, and (iii) complete replacement of wheat flour with oilcake flour combined with increasing Sacha inchi oil content. This addition clarifies the experimental

design and allows readers to better understand the progressive incorporation of Sacha inchi ingredients across formulations. The revised sentence now reads as follows:

> As summarized in **Table 1**, the formulations were designed to progressively increase the proportion of Sacha inchi ingredients, enabling the evaluation of both single and combined substitution effects. This resulted in three main substitution gradients: *(i)* replacement of sunflower oil with Sacha inchi oil (F1–F2); *(ii)* simultaneous 50 % substitution of wheat flour with oilcake flour and variable levels of oil (F3–F5); and *(iii)* total substitution of wheat flour with oilcake flour and increasing Sacha inchi oil content (F6–F8). The constant ingredients (egg, sugar, honey, salt, vanilla, and sodium bicarbonate) ensured that observed variations could be attributed primarily to differences in oil and flour composition.

Line 175 (Page 15): The energy calculation formula is correct, but the assumption of 4 kcal/g for protein and carbs and 9 kcal/g for fat should be explicitly stated.

**RESPONSE:** We thank the reviewer for this precise observation. In response, the text has been revised to explicitly indicate the conversion factors used in the energy value calculation. The following clarification has been added below Equation (2):

> Conversion factors of 9 kcal/g for fat and 4 kcal/g for protein and carbohydrates were applied according to the Atwater system [26].
>
> - Maclean WC, Harnly JM, Chen J, Chevassus-Agnes S, Gilani G, Livesey G, et al. Food energy—Methods of analysis and conversion factors. Rome: Food and Agriculture Organization of the United Nations.; 2003.

Line 203 (Page 16): The GC-MS quantification method assumes a 1:1 response factor—this should be justified or validated.

**RESPONSE:** We thank the reviewer for their valuable comment. In this revised version, the calibration curves have been included in the *Materials and Methods* section. These curves were constructed using three representative fatty acid standards—palmitic, oleic, and linoleic acids—purchased from Aldrich. As shown in the corresponding calibration plots, all standard curves exhibit excellent linearity ($R^2 > 0.99$) across the tested concentration ranges, with slopes close to unity. This high degree of linear correlation and slope consistency supports the assumption of a 1:1 response factor between analyte concentration and detector response. The revised section now reads as follows:

> For GC-MS analysis, a Shimadzu GCMS-QP-2010 Ultra Plus equipped with an AOC-20i/s autosampler and an autoinjector was used. The separation was performed on a DB-WAX gas chromatography (GC) capillary column (column thickness: 0.25 µm, length: 30.0 m, internal diameter: 0.25 µm). The oven configuration was set as follows: an initial temperature of 120 °C with a hold time of 2 min, followed by a ramp to 190 °C at 15 °C/min and a hold time of 4 min, then a ramp to 240 °C at 5 °C/min and a hold time of 10 min, and finally a ramp to 250 °C at 5 °C/min and a hold time of 3 min. The injector temperature was set at 250 °C, and 1 µL of the sample was injected with a 1:4 split mode. Helium was used as the carrier gas, considering the linear velocity for a column flow of 0.98. Mass detection was configured to start at an *m/z* of 35.00 and end at an *m/z* of 700.00. ~~To establish the calibration curves, individual fatty acid standards (concentration range: 1.45–2.55 mg/L) were used for palmitic, oleic, and linoleic acids.~~ Calibration curves were generated using individual standard solutions (1.45–2.55 mg/L) of palmitic, oleic, and linoleic acids. The corresponding

regression equations and analytical performance parameters were as follows: palmitic acid, $y = (0.9712 \pm 0.0119)x + (0.0377 \pm 0.0027)$, $R^2 = 0.9930$; oleic acid, $y = (0.9951 \pm 0.0086)x + (0.0245 \pm 0.0017)$, $R^2 = 0.9994$; and linoleic acid, $y = (0.9315 \pm 0.0144)x + (0.0442 \pm 0.0015)$, $R^2 = 0.9982$. ~~Fatty acid quantification was based on a 1:1 response factor, meaning that the mass ratio (mi/mt) was equivalent to the area ratio (Ai/At), where the mass fraction of each fatty acid was determined using the equation:~~ The mass ratio is “x”, and the area ratio is “y”. Based on the response factor of 1:1 for the previous three fatty acids (slope of about 1), which implies that mi/mt = Ai/At (mass ratio is equal to area ratio), it was possible to quantify the other fatty acids as:

$$\% \text{ Fai} = (\text{Ai}/\text{At}) \ x \ 100 \qquad (3)$$

where % Fai is the percentage of the corresponding fatty acid “i”, Ai is the area of the given fatty acid, and At is the total area (At = ΣAi). In the calibration curve equations, the mass ratio is “x”, and the area ratio is “y”. The results were expressed in grams of fatty acids per 100 g of oil (g/100 g for the oil).

Line 215 (Page 17): Recovery rates (84.70%–99.96%) are excellent—consider stating the acceptable range for validation.

**RESPONSE:** We thank the reviewer for this valuable observation. There is no universally fixed acceptable range for recovery values, as these depend on the analyte concentration, sample matrix, and analytical methodology employed. However, according to widely recognized validation guidelines—such as those established by the AOAC (*Appendix F: Guidelines for Standard Method Performance Requirements*) and the ICH (*Q2(R2): Validation of Analytical Procedures*)—recovery rates within 80–120% are generally considered acceptable for most analytical methods. Therefore, the recovery values obtained in this study (84.70–99.96%) fall well within these recommended limits, confirming the accuracy and reliability of the applied GC–MS quantification procedure.

- AOAC: https://www.aoac.org/wp-content/uploads/2019/08/app_f.pdf
- ICH: https://www.ema.europa.eu/en/ich-q2r2-validation-analytical-procedures-scientific-guideline

Line 229 (Page 18): The use of gallic acid equivalents is standard, but the calibration curve $R^2$ value should be reported.

**RESPONSE:** We thank the reviewer for this helpful observation. In the revised version, the calibration curve and $R^2$ information has been added to the *Materials and Methods* section. The sentence now reads as follows:

A standard curve was generated using gallic acid (30,6 - 102,1 mg/L), yielding the regression equation $y = 1,6509x + 0,007$ ($R^2 = 0,999$).

Line 269 (Page 19): The use of Tukey’s test is appropriate—ensure all comparisons in tables reflect this (some values lack superscripts).

**RESPONSE:** We thank the reviewer for this observation. We confirm that Tukey’s HSD test was indeed the appropriate post hoc analysis for our dataset, as it allows multiple pairwise comparisons while controlling the familywise error rate, ensuring a robust evaluation of mean differences among treatments. We acknowledge the unintentional omission of superscript letters in the fiber data and

have now corrected this by including the corresponding superscripts that indicate significant differences ($p < 0.05$) according to Tukey's test.

**Reviewer #2:** The study is quite well-prepared and detailed. However, a few points caught my attention. In the recipe for the cookie formulation, were the ingredients used on a dry basis? Secondly, why was no sensory evaluation performed? Including sensory evaluation could have been valuable, as it would allow the produced cookies to be assessed in terms of important criteria such as texture and consumer acceptability.

**RESPONSE:** We thank the reviewer for this observation. All ingredients were used in their natural state, as commercially available, without prior dehydration or concentration. Specifically, both flours (wheat flour and Sacha inchi oil cake flour) were used in their dry form, while Sacha inchi oil, sunflower oil, egg, vanilla flavor, sucrose (fine granulated), brownulated sucrose, honey, sodium bicarbonate, and salt were incorporated as received in their natural or liquid form according to standard baking practices. This clarification has been added to the Materials and Methods section as follows:

> All ingredients were used in their natural state, as commercially available, without prior dehydration or concentration. Wheat flour were used in their dry form, while Sacha inchi oil, sunflower oil, egg, vanilla flavor, sucrose (fine granulated), brownulated sucrose, honey, sodium bicarbonate, and salt were used as they are commercially sold or available in the marke.

**RESPONSE:** We also thank the reviewer for this valuable comment and fully agree that sensory evaluation provides important insights into product acceptability and consumer perception. In fact, a preliminary sensory evaluation was conducted as part of the broader experimental process to obtain indicative feedback on attributes such as appearance, texture, flavor, and overall acceptability. However, according to the national regulatory framework under which the study was performed, ethical committee approval is not required for non-invasive sensory tests involving food products that are safe for human consumption and do not pose any health risk. Nonetheless, PLOS ONE requires formal ethics committee approval for the publication of sensory data involving human participants, regardless of local exemptions. Given that such approval was not mandatory nationally and therefore was not obtained prior to data collection, we have opted not to include the sensory data in the present publication to ensure full compliance with PLOS ONE ethical policies. We wish to emphasize that this study was designed as an exploratory and technological assessment, primarily focused on the nutritional, physicochemical, and functional properties of the reformulated cookies. A subsequent phase of this research is already planned, in which a comprehensive sensory and consumer acceptance study will be conducted under formal ethical approval to complement and validate the present findings.

**Reviewer #3:** Structure the abstract into four distinct sections. For example, include a reference: https://doi.org/10.1371/journal.pone.0307506.

**RESPONSE:** We sincerely thank the reviewer for this valuable suggestion. In response to the recommendation, the abstract has been restructured following the PLOS ONE format, which includes the sections Background, Methodology, Results, and Conclusions. The revised abstract is now presented as follows:

**Background**
The increasing demand for healthier and more sustainable foods has driven the reformulation of bakery products using alternative ingredients. Sacha inchi (Plukenetia volubilis L.), an Amazonian oilseed rich in proteins, unsaturated fatty acids, and bioactive compounds, represents a promising resource to enhance the nutritional and functional quality of baked goods while valorizing agro-industrial by-products.
**Methodology**
This study investigated the effects of partially or totally replacing wheat flour and sunflower oil with defatted Sacha inchi oilcake flour and Sacha inchi oil on the nutritional, physicochemical, and functional properties of cookies. Nine formulations were developed, including a control and eight experimental variants. Proximate composition, mineral content, lipid profile, total polyphenols, antioxidant activity (FRAP assay), texture, geometry, and color parameters were evaluated.
**Results**
Cookies containing Sacha inchi flour showed significant increases in protein (up to 19.65%), fat, fiber (six-fold higher), ash, and energy, with a reduction in carbohydrates. Mineral content increased markedly for calcium (10.8-fold), magnesium (10.2-fold), potassium (3-fold), phosphorus (5.4-fold), and zinc (4.6-fold), while iron decreased by 25%. Lipid profiling revealed a higher proportion of polyunsaturated fatty acids, particularly α-linolenic acid (omega-3), and lower saturated fats in cookies containing Sacha inchi oil. The incorporation of Sacha inchi ingredients also increased total polyphenols (up to 1.46-fold) and antioxidant activity (up to 1.99-fold). Texture analysis showed that Sacha inchi flour reduced hardness (up to 5.8 times softer than control), whereas Sacha inchi oil increased firmness (up to 2.4 times). Full replacement of sunflower oil increased cookie diameter and spread ratio, while Sacha inchi flour reduced diameter and increased thickness. Color parameters were also affected, reflecting compositional and Maillard-related changes during baking.
**Conclusions**
Replacing wheat flour and sunflower oil with Sacha inchi flour and oil enhanced the nutritional profile, fatty acid composition, and antioxidant capacity of cookies, while modulating their texture and geometry. These findings demonstrate the technological feasibility of using Sacha inchi derivatives as functional ingredients in bakery products, supporting the development of sustainable, health-oriented foods and promoting the valorization of Amazonian crops and by-products.

Incorporate a flow diagram illustrating the cookie formulation process. For example, include a reference: https://doi.org/10.1371/journal.pone.0307506.

**RESPONSE:** We thank the reviewer for this valuable suggestion. In response, a flow diagram has been created and incorporated into the revised manuscript to visually summarize the entire workflow, including both the formulation and elaboration steps of the cookie production process. The new figure (now included as Figure 1) illustrates the sequential stages from ingredient preparation and formulation to mixing, dough formation, shaping, baking, and final storage. The following sentence has been added to the manuscript to reference this visual summary:

To enhance visualization and methodological clarity, a schematic flow diagram of the formulation and processing steps is provided in Figure 1.

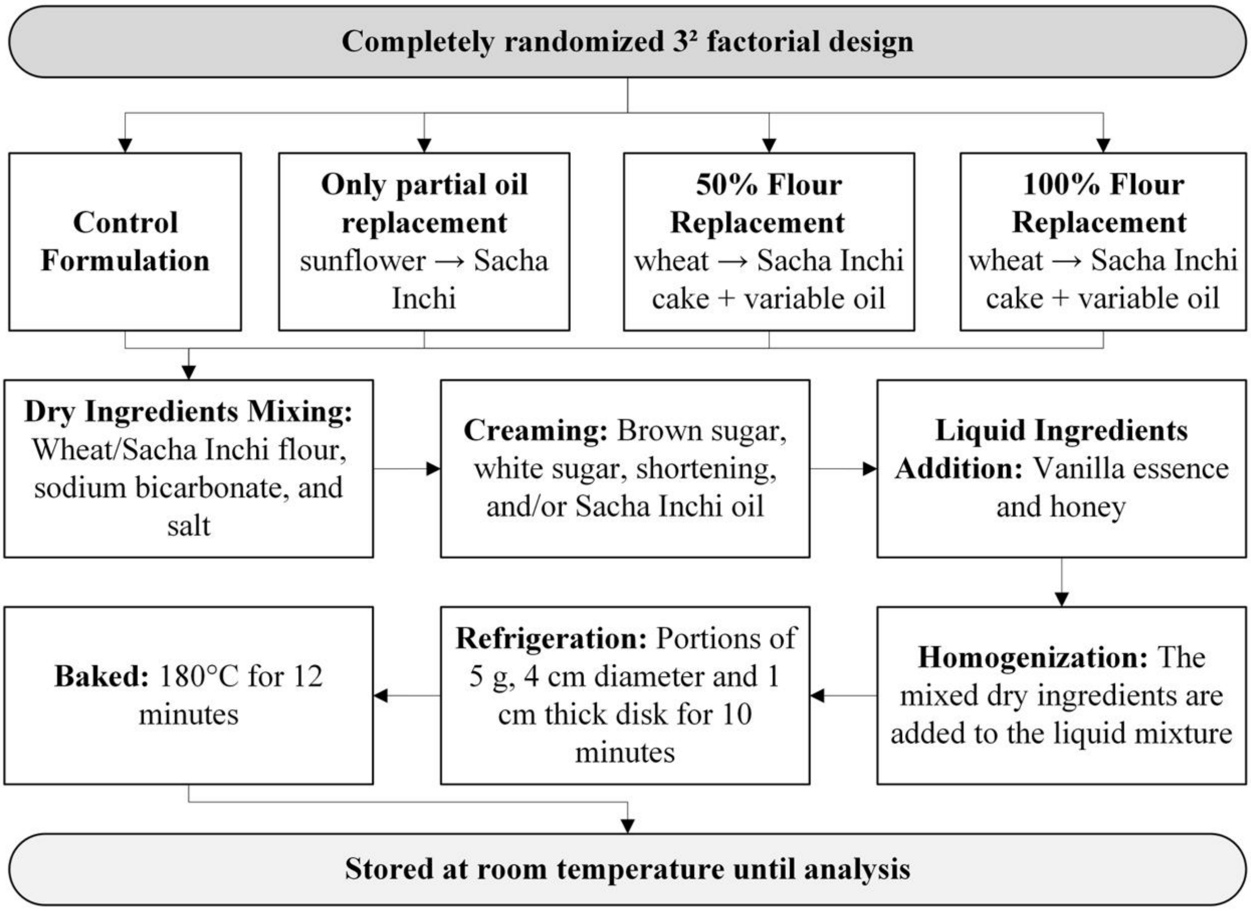


**Figure 1.** Flowchart illustrating the formulation and processing workflow of cookies enriched with Sacha inchi oil and/or defatted Sacha inchi cake flour, including ingredient preparation, mixing, dough formation, molding, and baking.

- Organize Table 1 following the format in the provided reference for clarity and ease of understanding: https://doi.org/10.1016/j.foohum.2025.100771.

**RESPONSE:** We thank the reviewer for this valuable suggestion. In response, Table 1 has been reformatted to follow, as closely as possible, the structure and style of the reference indicated, ensuring greater clarity and ease of interpretation. The revised version now separates the variable and fixed ingredients, highlighting the experimental factors under study while maintaining consistency with the data and scope presented in our manuscript.

Include the table and accompanying text in the main manuscript, as they are critical components. For example, adhere to the referenced format. Specify the equipment details (model, brand, country) in the Materials and Methods section.

**RESPONSE:** We appreciate the reviewer's observation. The data originally presented in the supplementary table correspond to the detailed chemical and bromatological characterization of the raw materials (defatted *Sacha inchi* cake flour and *Sacha inchi* oil) used in this study. These results have been previously analyzed and reported in a manuscript recently published by our research group

[17]. Unfortunately, due to the extended editorial process of the present article, that work was published first.
To avoid redundancy and potential duplication of published results, the supplementary materials have now been removed. Instead, we have incorporated the following clarifying statement in the *Materials and Methods* section, along with the appropriate citation:

> The chemical and bromatological composition of the raw materials employed in this study (defatted *Sacha inchi* cake flour and *Sacha inchi* oil) had been previously analyzed and fully characterized by our research group [17]. This prior characterization, conducted under standardized analytical protocols, established the compositional and physicochemical profile of both materials and confirmed their suitability for food formulation purposes. Consequently, both the oil and its defatted by-product were well-defined in terms of their nutritional and chemical attributes, providing a reliable and validated baseline for their use in the present study.


- Alejandro Ruiz FE, Ortega Jácome JF, Mora JR, Landázuri AC, Vásconez Duchicela P, Vásconez Espinoza J, et al. Comprehensive characterization and valorization potential of Amazonian Sacha inchi (Plukenetia volubilis L.) seeds, oil, and oilcake by-products for sustainable food applications. Front Nutr. 2025;12. doi:10.3389/fnut.2025.1597300


Provide the AOAC method number for each analytical determination.

**RESPONSE:** We thank the reviewer for this observation. Although the AOAC method numbers were already cited in the bibliography, we have now explicitly included the corresponding AOAC method number in the Methodology section, as follows:

> Proximate composition analyses of pre-ground cookies were conducted following the standardized protocols outlined in the AOAC Official Methods of Analysis. Color was evaluated using a Konica Minolta CR-400 colorimeter (Konica Minolta Sensing Americas, Inc., NY, EEUU) in the CIELAB color space [19]. Moisture content (%) was determined through gravimetric analysis (AOAC 925.10) [20], whereas water activity ($a_w$) was measured using a HygroLab C1 water activity meter (Rotronic, Grindelstrasse, CH-Switzerland). Ash content (%) was quantified by incinerating 3 g of samples in a muffle furnace at 550 ± 1 °C overnight (AOAC 923.03) [21]. Additionally, total crude fiber content (%) was assessed through sequential extraction with acidic and alkaline solutions (AOAC 978.10) [22] whyle the fraction corresponding to soluble (%) and insoluble fiber (%) using methods proposed by the Cereals & Grains Association (AACC 32-05.01) [23] and AOAC 985:29 [24]. Meanwhile, lipid content (%) was determined via Soxhlet extraction using petroleum ether as the solvent (AOAC 920:39) [25], whereas protein content (%) was analyzed using the Kjeldahl method, applying a nitrogen-to-protein conversion factor of 6.25 (AOAC 2001.11) [26].

Report the absorbance values used in spectrophotometric measurements, along with the quantification equation and the coefficient of determination.

**RESPONSE:** We thank the reviewer for this valuable observation and sincerely apologize for the unintentional omission. In this revised version, we have included, for each analytical method reported, the concentration range of the standards used, the corresponding calibration equation, and the coefficient of determination ($R^2$). These additions ensure a complete and transparent description of the spectrophotometric quantification procedures employed in this study.

Indicate the concentrations of solutions prepared in this section. Conduct a sensory analysis, as it is vital for this study.

**RESPONSE:** We thank the reviewer for this observation. As previously indicated, the concentrations of all standard solutions have been specified in the corresponding sections. We have also carefully reviewed the *Materials and Methods* section to ensure that the concentrations of all prepared solutions are clearly indicated wherever relevant and appropriate.

**RESPONSE:** We also fully agree that sensory evaluation represents a key component in assessing product acceptability and consumer perception. Indeed, a preliminary sensory assessment was carried out during the broader experimental process to gather indicative feedback on sensory attributes such as appearance, texture, flavor, and overall acceptability. However, under the national regulatory framework applicable to this research, ethical committee approval is not required for non-invasive sensory evaluations involving food products that are safe for human consumption and pose no health risks. Nevertheless, *PLOS ONE* requires formal ethical approval for any study involving human participants, including sensory assessments, regardless of local exemptions. Since such approval was not mandatory at the national level and was therefore not sought prior to data collection, we have opted not to include the sensory data in the current manuscript to ensure full adherence to *PLOS ONE*'s ethical publication policies.
We would like to emphasize that this study was primarily designed as a technological and analytical assessment focused on the nutritional, physicochemical, and functional properties of the reformulated cookies. A follow-up phase of this research is already planned, which will include a comprehensive sensory and consumer acceptance study conducted under formal ethical approval to complement and validate the present findings.

Include references in the methodology section for procedures such as color determination and hardness measurements.

**RESPONSE:** We appreciate the reviewer's observation. As requested, we have included the corresponding references in the Methodology section for the procedures concerning color determination and hardness measurements.

- Kuchtová, V., Kohajdová, Z., Karovičová, J., & Lauková, M. (2018). Physical, textural and sensory properties of cookies incorporated with grape skin and seed preparations. Polish Journal of Food and Nutrition Sciences, 68(4), 309-317.
- Alp Baltakesmez D, Mısır S. Determination of various physical, structural properties and sensory acceptability of Hatay Kömbe cookies produced from Ancestral Seed Kavılca (Triticum dicoccum L.). Harran Tarım ve Gıda Bilimleri Dergisi. 2025;29: 177–190. doi:10.29050/harranziraat.1572194

In Table 2, report either moisture content or water activity, as including both is unnecessary.

**RESPONSE:** We appreciate the reviewer's suggestion. However, we believe it is scientifically important to retain both moisture content and water activity (aw) in Table 2, as they describe distinct yet complementary aspects of the product's physicochemical state. Moisture content quantifies the total amount of water (free + bound) in the matrix, which is critical for evaluating formulation differences, process efficiency, and nutritional composition. In contrast, water activity reflects the availability of free water for chemical and microbial processes, directly influencing shelf-life stability, microbial safety, texture, and lipid oxidation.

In bakery systems such as cookies, these two parameters are not linearly correlated because composition (protein, fiber, sugar, and fat levels) and processing conditions affect how water is bound or retained. For instance, in our formulations, Sacha inchi oil and oilcake flour altered the matrix structure so that moisture varied slightly while aw decreased markedly, demonstrating that the two indicators convey different information. Reporting both values therefore provides a more complete understanding of water distribution and stability mechanisms, supporting a robust interpretation of texture and storage behavior. For these reasons, we consider the inclusion of both parameters justified and scientifically valuable for reproducibility and comparison with related studies.
