# Supplementary material for: Influence of partial or total substitution of wheat flour and sunflower oil with Sacha inchi (Plukenetia volubilis L.) flour and oil on the quality and nutritional properties of cookies
Source: PLoS One. 2026 Jan 6;21(1):e0340190. doi: 10.1371/journal.pone.0340190 (PMC12774371; doi:10.1371/journal.pone.0340190)
Supplement: Attachment — Submitted filename: Review PONE-D-25-18191R1.docx [file pone.0340190.s003.docx]

**Summary – Manuscript PONE-D-25-18191R1**

The present work investigates Sacha inchi (Plukenetia volubilis L.), a plant native to the Amazon basin, for its role as a primary ingredient in the process of baking cookies for the purpose of sustainable, health conscious, large scale food production. The work suggests that Sacha inchi, which is a rare seed oil, increases the macronutrient composition of cookies in favor of a more healthy nutritional profile. Given rising concerns with prevalence of metabolic disorder, dysbiosis arising from overconsumption of highly-processed foods, and an expanding agricultural/environmental dilemma from current practices, this is a timely and important study looking to shift the paradigm of industrial processes with a basis in indigenous nutritional practices immemorial. This work is supported by a body of literature that has previously described the nutrient composition of Sacha inchi, which is rich in omega-3 fatty acids and its refined by-products are similarly rich in nutritive chemicals; the authors astutely point to some of the undesired by-products and the current processes of removal and refinement that render these to be relatively safe. Sacha inchi and its by-products were used as replacements for wheat flour and sunflower oil, then products were generated by modulating the amounts of Sacha inchi products in the formulation - materials and products were evaluated for nutrient content, lipid composition, polyphenol and antioxidant activities, and physical properties. The study is promising for shifting the diversity of nutritional sources in large-scale food processing and leverages existing ingredients with presence in the historical lexicon. The study is limited by its evaluation of only the products at end-stage and using a single baking method, which may require refinement specific to the individual compositions. The expected benefits of each of Sacha inchi’s components are well-described, but the combination of these nutritional factors, their chemical interactions, and their potential bioavailability remain unknown. As well, the physical parameters suggest a subjective score representing desirability of the final product, however the roles for taste and texture in the eating experience are not described, as this would have included a human subjects component. This supports future studies looking into subjective experience using optimized formulations with human cohorts, and does not reduce enthusiasm for this report. Please see my detailed comments.

Major Comments:

Evaluation of lipid composition is a strength of this work, however further dissection of omega-3 and omega-6 lipid species (e.g., docosahexaenoic acid, eicosapentaenoic acid, arachidonic acid, etc.) and short chain fatty acids would further strengthen this work.

Subjective response to the various cookie formulations would provide additional rationale for the physical outcomes measured. As well, features such as taste and texture (beyond rigidity) are not described, and will play a major role with incorporating Sacha inchi into regular use.

Minor Comments:

The results section of the abstract indicates fold change for large effects and percentage values for smaller effects. This may be made more consistent by using the percentage notation throughout but is acceptable. “Six-fold” higher should be rewritten with the numerals, “6-fold”, to match the subsequent effects sizes (i.e., 10.8-fold, 10.2-fold, etc.).

The claim in Lines 101-103, “…traditionally consumed by indigenous communities for their health benefits and nutrient density.”, require additional citation. Are there accounts of the particular ingredients, described herein, being used medicinally?
