# Supplementary material for: Influence of partial or total substitution of wheat flour and sunflower oil with Sacha inchi (Plukenetia volubilis L.) flour and oil on the quality and nutritional properties of cookies
Source: PLoS One. 2026 Jan 6;21(1):e0340190. doi: 10.1371/journal.pone.0340190 (PMC12774371; doi:10.1371/journal.pone.0340190)
Supplement: Attachment — Submitted filename: Responses to reviewers.docx [file pone.0340190.s005.docx]

**Reviewer 1**

In view of this manuscript, the data given about Sacha inch is not sufficient. Give some detail about this composition, etc.

**Response:** We appreciate the reviewer’s observation and agree that additional compositional detail strengthens the contextualization of Sacha inchi within the introduction. Accordingly, we have expanded this section by incorporating a concise summary of its key nutritional and chemical attributes. The revised text now reads as follows:

Sacha inchi is recognized for its exceptional chemical composition, with seeds containing 33–54% oil, 24–30% protein, and an optimal ω-6/ω-3 ratio close to 1:1, driven by high levels of α-linolenic acid (∼47%) and linoleic acid (∼38%) [13]. Recent characterization of Ecuadorian materials confirms this fatty acid profile and additionally shows that the defatted oilcake is highly concentrated in protein (~42%), dietary fiber, and essential minerals, including calcium (2,63 mg/kg), magnesium (3,69 mg/kg), potassium (7,68 mg/kg), and phosphorus (9,14 mg/kg), while retaining meaningful levels of polyphenols and antioxidant activity [14]. These compositional attributes, together with favorable techno-functional properties such as water absorption and swelling capacity, support the use of both Sacha inchi oil and oilcake flour as nutrient-dense, sustainable ingredients for bakery formulations.

Some common mistakes like incorrect spellings, grammatical mistakes are also present in this manuscript. Kindly correct all these.

**Response:** We thank the reviewer for pointing this out. The entire manuscript has now been thoroughly revised for language quality, grammar, spelling, and academic style. A professional native-English proofreader has reviewed and corrected the full text to ensure clarity, consistency, and linguistic accuracy.

In GC MS Analysis, there is no citation reference is given in overall paragraph.

**Response:** We appreciate the reviewer’s observation. We would like to clarify that the methodology is indeed properly cited in the paragraph immediately preceding the GC–MS description. Specifically, the manuscript states:

*“Fatty acid content analysis was performed following the internal standard method described by Ponce et al. and based on AC Analytical Controls Application Note 1410 [32,33].”*

These references cover the analytical procedure used and provide the methodological foundation for the GC–MS analysis presented. Nonetheless, we have reviewed this section to ensure that the citation placement is clear and unambiguous.

Again, there is no citation is given in color determination method, hardness determination and statistical analysis. Give proper citation in overall manuscript.

**Response:** We thank the reviewer for this observation and sincerely apologize for the unintentional omission of the corresponding citations in the sections describing the color determination and hardness determination methods. In the revised manuscript, we have now added the appropriate methodological references to ensure proper attribution and methodological accuracy.

Regarding the statistical analysis, we would like to clarify that the manuscript already includes a dedicated subsection titled “Design of Experiment and Statistical Analysis” within the Materials and Methods section, where all statistical procedures, software, and analytical criteria are fully described. We have verified that this section clearly communicates the statistical framework applied in our study.

In addition, we have thoroughly reviewed the entire manuscript to ensure that all methodological sections contain their complete and correct references, and to confirm that no methodological citation is missing in the revised version.

Please write all references according to Journal guidelines in Vancouver style, mistakes like non abbreviated journals names, space issues etc are present, deeply attention is required in this regard.

**Response:** We thank the reviewer for highlighting this issue. All references in the manuscript have now been thoroughly revised and corrected to fully comply with the journal’s Vancouver style guidelines, including proper journal name abbreviations, spacing, punctuation, and overall formatting. We have carefully checked the entire reference list and all in-text citations to ensure complete consistency with the required style.

**Reviewer 4**

**Summary – Manuscript PONE-D-25-18191R1**

The present work investigates Sacha inchi (Plukenetia volubilis L.), a plant native to the Amazon basin, for its role as a primary ingredient in the process of baking cookies for the purpose of sustainable, health conscious, large scale food production. The work suggests that Sacha inchi, which is a rare seed oil, increases the macronutrient composition of cookies in favor of a more healthy nutritional profile. Given rising concerns with prevalence of metabolic disorder, dysbiosis arising from overconsumption of highly-processed foods, and an expanding agricultural/environmental dilemma from current practices, this is a timely and important study looking to shift the paradigm of industrial processes with a basis in indigenous nutritional practices immemorial. This work is supported by a body of literature that has previously described the nutrient composition of Sacha inchi, which is rich in omega-3 fatty acids and its refined by-products are similarly rich in nutritive chemicals; the authors astutely point to some of the undesired by-products and the current processes of removal and refinement that render these to be relatively safe. Sacha inchi and its by-products were used as replacements for wheat flour and sunflower oil, then products were generated by modulating the amounts of Sacha inchi products in the formulation - materials and products were evaluated for nutrient content, lipid composition, polyphenol and antioxidant activities, and physical properties. The study is promising for shifting the diversity of nutritional sources in large-scale food processing and leverages existing ingredients with presence in the historical lexicon. The study is limited by its evaluation of only the products at end-stage and using a single baking method, which may require refinement specific to the individual compositions. The expected benefits of each of Sacha inchi’s components are well-described, but the combination of these nutritional factors, their chemical interactions, and their potential bioavailability remain unknown. As well, the physical parameters suggest a subjective score representing desirability of the final product, however the roles for taste and texture in the eating experience are not described, as this would have included a human subjects component. This supports future studies looking into subjective experience using optimized formulations with human cohorts, and does not reduce enthusiasm for this report. Please see my detailed comments.

Major Comments:

- Evaluation of lipid composition is a strength of this work, however further dissection of omega-3 and omega-6 lipid species (e.g., docosahexaenoic acid, eicosapentaenoic acid, arachidonic acid, etc.) and short chain fatty acids would further strengthen this work.

**Response:** We appreciate the reviewer’s suggestion regarding the need to further clarify the presence or absence of specific omega-3 and omega-6 lipid species, as well as short-chain fatty acids. In response, we have incorporated the following paragraph into the Fatty acid profile and content of cookies formulated with Sacha inchi flour and oil section:

*“No long-chain omega-3 fatty acids (such as EPA and DHA) or long-chain omega-6 fatty acids (e.g., arachidonic acid) were detected in any of the formulations. This is consistent with the previously reported fatty acid profile of Sacha inchi oil, which is predominantly α-linolenic acid (C18:3, ω-3) together with linoleic acid (C18:2, ω-6), and lacks long-chain omega-3 PUFA such as EPA and DHA. Similarly, no short-chain fatty acids (C4:0–C10:0) were observed in our chromatograms, which is also consistent with previous reports for oils from this seed [8,17].”*

This addition clarifies the absence of these fatty acids in our formulations and aligns our findings with previously reported compositional data for Sacha inchi oil.

- Subjective response to the various cookie formulations would provide additional rationale for the physical outcomes measured. As well, features such as taste and texture (beyond rigidity) are not described, and will play a major role with incorporating Sacha inchi into regular use.

**Response:** We appreciate the reviewer’s observation regarding the value of incorporating sensory or subjective evaluations to complement the physical and compositional results. We fully agree that sensory perception—including taste, aroma, mouthfeel, and overall acceptability—plays a crucial role in determining the practical relevance and consumer integration of new formulations containing Sacha inchi.

However, conducting any form of sensory evaluation, even at an exploratory level, requires prior approval from an accredited institutional ethics committee, as human participation is involved. Unfortunately, ethical approval for sensory testing was not included in the scope of the original study design. In accordance with PLOS ONE’s policies, the inclusion of human sensory data is strictly contingent upon documented approval by an institutional review board or ethics committee. Because this approval was not obtained beforehand, we are unable to incorporate sensory evaluation results in the current manuscript.

We would also like to note that this point was raised by previous reviewers, who fully understood our explanation regarding the ethical limitations and the impossibility of adding sensory data at this stage. We respectfully hope that the current reviewer will similarly appreciate the rationale for the absence of sensory analyses in this study.

Minor Comments:

- The results section of the abstract indicates fold change for large effects and percentage values for smaller effects. This may be made more consistent by using the percentage notation throughout but is acceptable. “Six-fold” higher should be rewritten with the numerals, “6-fold”, to match the subsequent effects sizes (i.e., 10.8-fold, 10.2-fold, etc.).

**Response:** We thank the reviewer for this helpful observation regarding consistency in numerical notation within the abstract. In accordance with the suggestion, we have revised the expression “six-fold higher” to “6-fold higher” to ensure uniformity with the subsequent effect sizes reported (e.g., 10.8-fold, 10.2-fold, 3-fold). We have also harmonized fold-change terminology throughout the abstract to improve clarity and consistency. The revised abstract now reflects a coherent numerical format.

*“Cookies containing Sacha inchi flour showed significant increases in protein (up to 19.65%), fat, fiber (6-fold higher), ash, and energy, with a reduction in carbohydrates. Mineral content increased markedly for calcium (10.8-fold), magnesium (10.2-fold), potassium (3-fold), phosphorus (5.4-fold), and zinc (4.6-fold), while iron decreased by 25%. Lipid profiling revealed a higher proportion of polyunsaturated fatty acids, particularly α-linolenic acid (omega-3), and lower saturated fats in cookies containing Sacha inchi oil. The incorporation of Sacha inchi ingredients also increased total polyphenols (up to 1.46-fold) and antioxidant activity (up to 1.99-fold). Texture analysis showed that Sacha inchi flour reduced hardness (up to 5.8-fold softer than the control), whereas Sacha inchi oil increased firmness (up to 2.4-fold). Full replacement of sunflower oil increased cookie diameter and spread ratio, while Sacha inchi flour reduced diameter and increased thickness. Color parameters were also affected, reflecting compositional and Maillard-related changes during baking*.”

- The claim in Lines 101-103, “…traditionally consumed by indigenous communities for their health benefits and nutrient density.”, require additional citation. Are there accounts of the particular ingredients, described herein, being used medicinally?

**Response:** We thank the reviewer for this important clarification request. In response, we have added an appropriate citation documenting the traditional consumption and reported health-related uses of Sacha inchi among Indigenous communities. This reference has now been incorporated into the revised manuscript to support the statement regarding its traditional relevance and perceived benefits.

*Rios M, Koziol MJ, Borgtoft Pedersen H, Granda G, editors. Plantas Útiles del Ecuador: Aplicaciones, Retos y Perspectivas / Useful Plants of Ecuador: Applications, Challenges, and Perspectives. Quito: Ediciones Abya-Yala; 2007.*

*Cárdenas Sierra DM, Gómez Rave LJ, Soto JA. Biological Activity of Sacha Inchi (Plukenetia volubilis Linneo) and Potential Uses in Human Health: A Review. Food Technol Biotechnol. 2021;59: 253–266. doi:10.17113/ftb.59.03.21.6683*
